# Adipose Tissue Myeloid-Lineage Neuroimmune Cells Express Genes Important for Neural Plasticity and Regulate Adipose Innervation

**DOI:** 10.3389/fendo.2022.864925

**Published:** 2022-06-20

**Authors:** Magdalena Blaszkiewicz, Gilian Gunsch, Jake W. Willows, Miranda L. Gardner, Jesse A. Sepeda, Andrew R. Sas, Kristy L. Townsend

**Affiliations:** ^1^Neurobiology and Energy Balance Laboratory, Department of Neurological Surgery, The Ohio State University, Wexner Medical Center, Columbus, OH, United States; ^2^Department of Cancer Biology and Genetics, Comprehensive Cancer Center, The Ohio State University, Wexner Medical Center, Columbus, OH, United States; ^3^Campus Chemical Instrument Center, Mass Spectrometry and Proteomics Facility, The Ohio State University, Columbus, OH, United States; ^4^Department of Neurology, The Ohio State University, Wexner Medical Center, Columbus, OH, United States

**Keywords:** scWAT, BDNF, energy expenditure, neuroimmune, neural plasticity, CINCs, cold stimulation, adipose neuropathy

## Abstract

Peripheral nerves allow a bidirectional communication between brain and adipose tissues, and many studies have clearly demonstrated that a loss of the adipose nerve supply results in tissue dysfunction and metabolic dysregulation. Neuroimmune cells closely associate with nerves in many tissues, including subcutaneous white adipose tissue (scWAT). However, in scWAT, their functions beyond degrading norepinephrine in an obese state remain largely unexplored. We previously reported that a myeloid-lineage knockout (KO) of brain-derived neurotrophic factor (BDNF) resulted in decreased innervation of scWAT, accompanied by an inability to brown scWAT after cold stimulation, and increased adiposity after a high-fat diet. These data underscored that adipose tissue neuroimmune cells support the peripheral nerve supply to adipose and impact the tissue’s metabolic functions. We also reported that a subset of myeloid-lineage monocyte/macrophages (Ly6c+CCR2+Cx3cr1+) is recruited to scWAT in response to cold, a process known to increase neurite density in adipose and promote metabolically healthy processes. These cold-induced neuroimmune cells (CINCs) also expressed BDNF. Here we performed RNAseq on CINCs from cold-exposed and room temperature-housed mice, which revealed a striking and coordinated differential expression of numerous genes involved in neuronal function, including neurotrophin signaling and axonal guidance, further supporting that CINCs fulfill a nerve-supporting role in adipose. The increased expression of leukocyte transendothelial migration genes in cold-stimulated CINCs also confirms prior evidence that they are recruited to scWAT and are not tissue resident. We now provide whole-depot imaging of scWAT from LysM-BDNF KO mice, revealing a striking reduction of innervation across the depot fitting with their reduced energy expenditure phenotype. By contrast, Cx3cr1-BDNF KO mice (a macrophage subset of LysM+ cells) exhibited increased thermogenesis and energy expenditure, with compensatory increased food intake and no change in adiposity or body weight. While these KO mice also exhibit a significantly reduced innervation of scWAT, especially around the subiliac lymph node, they displayed an increase in small fiber sympathetic neurite branching, which may underlie their increased thermogenesis. We propose a homeostatic role of scWAT myeloid-lineage neuroimmune cells together in nerve maintenance and neuro-adipose regulation of energy expenditure.

## Introduction

Adipose tissue innervation by the peripheral nervous system is demonstrably required for proper tissue function and metabolic homeostasis (1, 2) as was revealed by numerous tissue denervation studies (chemical and surgical) over the past decades. We have more recently described situations of “adipose neuropathy” (or reduction of neurite density, which is likely due to peripheral tissue axon die-back) with obesity, diabetes, and aging (3), which fits with the known loss of proper innervation of skin and muscle in these metabolic and neuropathic states. The peripheral nerves, both sensory and sympathetic, that innervate subcutaneous white adipose tissue (scWAT) are plastic, similar to what has been described extensively in the peripheral sciatic nerve (4–6), that is, peripheral nerves are capable of regrowth and axon regeneration as has been demonstrated after a sciatic nerve crush (7–9). As such, denervation of adipose tissue is transient, followed by re-innervation after 3 to 4 weeks, depending on the neurotoxin administered, and adipose neurites can increase in density after cold stimulation (3, 10). In addition, adipose tissue innervation is incredibly heterogeneous and diverse: the patterns of innervation differ in the anterior *versus* posterior regions of the inguinal depot, with a higher innervation observed around the lymph nodes, a mix of myelinated/non-myelinated fibers, thin axons innervating the parenchymal cells as well as the blood vessels, and branching thick nerve bundles (11). Therefore, it is important to gain a better understanding of the tissue factors that regulate adipose innervation status, plasticity, and the patterns of nerve distribution given that adipose tissues can undergo numerous fluctuations in adipose mass that would require control and remodeling of the nerve supply. This unique feature of adipose tissue expandability also makes it a particularly relevant tissue to investigate peripheral nerve dynamics and their support by local tissue factors.

We previously discovered that the deletion of brain-derived neurotrophic factor (BDNF) from the myeloid lineage (using LysM-Cre) resulted in a “genetic denervation” of scWAT, with a blunted browning response to cold stimulation and increased adipose mass after high-fat diet (12). These data underscored the importance of adipose nerves in these metabolic states of negative energy balance (cold) or positive energy balance (obesity). Single-nucleotide polymorphisms in BDNF and its receptor TrkB are associated with obesity in humans (13–15), and BDNF is clearly involved in energy balance regulation both in the brain (where it impacts appetite and energy expenditure) as well as in the periphery where BDNF is found circulating in blood and also locally produced in tissues, such as adipose tissue stromal vascular fraction (SVF) cells (16). After cold stimulation, we also previously conducted a comprehensive characterization of adipose tissue immune cell phenotypes and discovered a novel myeloid-lineage monocyte/macrophage marked by Ly6c+Ccr2+Cx3cr1+ that infiltrated scWAT after cold in both male and female mice, which we named cold-induced neuroimmune cells (CINCs) given their expression of BDNF (12). Given their markers, these are likely inflammatory and pro-regenerative immune cells that are recruited to scWAT by CCL2/MCP1. Neuroimmune cells are found in association with nerves in various tissues and engage in a bidirectional crosstalk between the nervous system in order to maintain tissue function and homeostasis. Several other neuroimmune cells have been described in adipose tissues, including sympathetic-associated macrophages (SAMs) that can carry and degrade norepinephrine in an obese state (17). Notably, a commonality shared by neuroimmune cells identified in adipose is the expression of Cx3rc1 (12, 17–19).

In the current study, we build on our previous findings and have now accomplished the following: (1) conducted an unbiased gene expression analysis of CINCs with and without cold stimulation to reveal the coordinated upregulation of gene expression networks related to nerve maintenance and plasticity, (2) imaged TrkB+ nerves in adipose, CINC localization in scWAT nerves and lymphatics, and putative nerve terminals in association with SVF cells, and (3) assessed the involvement of adipose neuroimmune cells in providing tissue neurotrophic factor in both the LysM-BDNF knock-out model as well as a new Cx3cr1-BDNF knock-out (a macrophage subset of the LysM myeloid lineage). Our data together reveal that adipose tissue neuroimmune cells are important for the maintenance of adipose innervation and metabolic function.

## Materials and Methods

### Mice, Metabolic Phenotyping, and *In Vivo* Analyses

#### Animals

The following mouse strains were obtained from The Jackson Laboratory: C57BL/6J (stock #000664), LysMCre+/-(B6.129P2-Lyz2/J, stock #004781), BDNF^fl/fl^ (Bdnf^tm3Jae^/J, stock #004339), R26R-EYFP [B6.129X1-Gt(ROSA)26Sortm1(EYFP)Cos/J, stock #006148], Cx3cr1CreER [B6.129P2(C)-Cx3cr1tm2.1(cre/ERT2)Jung/J, stock #020940], Ccr2^RFP^Cx3cr1^GFP^ Dual Reporter [B6.129(Cg)-Cx3cr1tm1Litt Ccr2tm2.1Ifc/JernJ, stock #032127], and TrkB^GFP^ [B6.129S6(Cg)-Ntrk2<tm2.1Ddg>/J, stock #023046]. LysM reporter mice were generated by crossing LysMCre^+/-^ *×* R26R-EYFP mice. Animals with myeloid-specific deletion of BDNF (*LysMCre^+/-^:: BDNF^-/-^)* or macrophage subset-specific deletion of BDNF (*Cx3cr1Cre^+/-^::BDNF^-/-^)* were generated in our facility by crossing Cx3cr1Cre transgene mice with BDNF^fl/fl^ mice, resulting in a F1 generation of BDNF BDNF^fl/wt^. This generation was further intercrossed, yielding Cx3cr1Cre*^+/-^::*BDNF^-/-^ mice. For both *LysMCre^+/-^::BDNF^-/-^* and *Cx3cr1Cre^+/-^::BDNF^-/-^* models, only F2 mice were used for all experiments due to a previously observed loss of phenotype in F5 *LysMCre^+/-^::BDNF^-/-^* mice. Monocytes/macrophage Dual Reporter (Ccr2^RFP^Cx3cr1^GFP^) mice and TrkB^GFP^ reporter mice were maintained heterozygous, and only heterozygous mice were used for the experiments. The animals were housed at 4 to 5 individuals in a cage, providing for socialization, in a temperature-monitored and humidity-controlled environment with 12/12-h light/dark cycle. The mice had *ad libitum* access to food and water, and the cages were replaced weekly. For all studies, the animals were euthanized using CO_2_, followed by cervical dislocation. To induce gene recombination in Cx3cr1creER mice, tamoxifen (at 100 mg/kg body weight) was administered *via* i.p. injections for 4 to 5 consecutive days as previously described (20). All experimental assessments began at 7 days after the last injection. Briefly, a 5-ml stock solution of 20 mg/ml tamoxifen (Sigma, catalog #T5648) was prepared fresh daily by dissolving 100 mg of tamoxifen powder in 500 ul of ethanol and then diluting in corn oil. The mixture was vortexed vigorously and placed in a shaking water bath overnight at 37°C to achieve the full solubilization of tamoxifen.

#### CLAMS Metabolic Cage Assessments

Physiological phenotyping in a metabolic cage was conducted in a Comprehensive Laboratory Animal Monitoring System (CLAMS; Columbus Instruments, Columbus, OH, USA) as previously described (12) for adult (at least 12 weeks old) male mice [*LysMCre^-/-^::BDNF^fl/fl^* (Con) and *LysMCre^+/-^::BDNF^-/-^* (KO) as well as *Cx3cr1^-/-^::BDNF^fl/fl^* (Con) and *Cx3cr1Cre^+/-^::BDNF^-/-^* (KO)]. Briefly, for all assessments, the animals were single-housed in a bedding-free cage at room temperature and on a 12-h light/dark cycle. Measurements of oxygen consumption (VO_2_) and carbon dioxide production (VCO_2_) were obtained every 15 min for 72 h (the first 24 h were used for acclimation) from which both the respiratory exchange ratio (RER) and the energy expenditure (heat) were calculated: RER = VCO_2_/VO_2_; energy expenditure (heat) = CV * VO_2_ cal/h, where CV is the “caloric value” as given by CV = (3.815 + 1.232) * RER. The waveform analysis of CLAMS data was performed by matching every 15-min measurement across two 24-h cycles. An ordinary two-way analysis of variance (ANOVA) was performed for average VO_2_, VCO_2_, RER, and heat per group. An uncorrected Fisher’s least significance difference test was performed for each timepoint between the Con and KO animals. Main effect *P*-values are reported.

#### Body Composition and Food Intake

The body composition of adult 12–14-week-old *LysMCre^-/-^::BDNF^fl/fl^* (Con) *and LysMCre^+/^::BDNF^-/-^* (KO) as well as *Cx3cr1^-/-^::BDNF^fl/fl^* (Con) *and Cx3cr1Cre^+/-^::BDNF^-/-^* (KO) male mice was measured using the EchoMRI™ 3-in1 analyzer (EchoMRI, LLC.)

#### Cold Exposure

Cold exposure was carried out in a diurnal incubator (Caron, Marietta, OH, USA) at 5°C and a 12-h light/dark cycle. The animals were housed at 1 to 2 in a cage and continuously cold-exposed for 3–10 days depending on the experimental design.

#### Thermal Imaging and Thermography Quantification

*In vivo* thermal imaging was obtained using a FLIR T560 Thermal camera (Teledyne FLIR. LLC). The animals were placed on the grid top of a cage lid, the fur was slicked away from the skin with olive oil, and the animal was held in place by its tail. Lateral side imaging (to assess the inguinal scWAT area) was performed at a distance of 0.42 m for all animals. The thermal images were analyzed using FLIR Research Studio Application. For each image, a region of interest (ROI; equal for all animal images within a cohort) was selected to cover the flank area (representing the inguinal scWAT region), and the mean temperature for the ROI was recorded. Per animal, *n* = 3 images were analyzed using FLIR Research Studio Application, and the average for each animal is graphed in GraphPad Prism.

### SVF Isolation and Fluorescence-Activated Cell Sorting

SVF from bilateral whole inguinal adipose depots was isolated as previously described (12). Briefly, the tissue was minced in 37°C pre-warmed Dulbecco’s modified Eagle’s medium (DMEM; high in glucose and serum-free) containing 2 mg/ml Roche Collagenase A (Millipore-Sigma, St. Louis, MO, USA; catalog #10103586001) at a volume of 10 ml/depot. The minced tissue in DMEM/collagenase was placed in a 50-ml conical tube and transferred to a shaking water bath at 37°C. The cells were dispersed by vortex and pipette mixing every 10 min. Full dissociation was achieved within 2 h. Media with dissociated tissue was filtered through 100-um cell strainers, rinsed with DMEM, and centrifuged at 500*g* for 10 min to separate adipocytes from SVF. The SVF pellet was incubated with 500 ul of RBC lysis buffer on ice for 2 min; 2 ml of DMEM with serum was added to stop the lysis. The cells were centrifuged at 500*g* for 5min at 4°C and resuspended in FACS buffer for cell sorting. For cell sorting, the following 5-marker panel was used with 4′,6-diamidino-2-phenylindole (DAPI) exclusion for viability: anti-mouse Ly6C_BV570 (HK1.4), anti-mouse CD11b PE (M1/70), anti-mouse CX3CR1 PercP5.5, SA011F11), anti-mouse CD45-PE-Cy7 (30-F11), and anti-mouse CCR2 A700 (475301). Sorting was performed on a BD™ FACSAria II™ cell sorter with SVF gated on CD45 and CD11b. CD45+CD11b- represented the non-myeloid population. The CD45^+^CD11b^+^ myeloid fraction was gated on Ly6C, followed by CCR2 and Cx3CR1. The sorted cells were collected in RLT buffer supplemented with beta-mercaptoethanol, vortexed, and snap-frozen for RNA extraction.

### RNA Extraction and Sequencing

RNA extraction and sequencing were performed by the Jackson Laboratory’s Genomics Core (Bar Harbor, ME, USA). RNA was extracted using the RNeasy Micro kit (Qiagen, catalog #74004). For the library construction (total RNA, rRNA depleted with no selection for polyA), a low-input RNA-seq kit (Takara SMARTer Stranded Total RNA-Seq v2 kit) was used. Quality control for the libraries was performed using Kapa qPCR for Illumina and the Agilent Bioanalyzer or TapeStation. The libraries were pooled and sequenced on two 1 × 75-bp NextSeq High Output sequence runs. The pooled libraries will be demultiplexed, and fastq files were made available. All samples were subjected to quality control analysis by NGSQCToolkit. Sample reads over 70 nucleotides long (75-bp reads) with a base quality score ≥30 were retained for further analysis. Read alignment and expression estimation were performed using RSEM v 1.2.12 with supplied annotations at default parameters against the mouse genome (build-mm10).

### RNAseq Data Processing, Differential Expression, and Pathway Analyses

Single-end, Illumina-sequenced stranded RNA-Seq raw count files (*n* = 10) were merged into a single datasheet by Ensembl identifier (Ensembl ID) and filtered to retain genes with counts >15 and in at least 3 observations of one group. The filtered reads were aligned to *Mus musculus* GRCm38 using RSEM (v1.2.12) to launch Bowtie2 (v2.2.0) (command: rsem-calculate-expression -p 12 –phred33-quals –seed-length 25 –forward-prob 0 –time –output-genome-bam – bowtie2 –paired-end). Potential outliers (C3, C5, RT1, and RT4b) were identified by performing an unbiased hierarchical clustering with hclust function from stats package in R, setting the method to “ward.d”, and excluded from downstream informatic analyses. The Ensembl ID for the remaining samples (*N* = 6) was converted to EntrezID with the select function in bioMaRt package, retaining only genes with converted EntrezID entries. Counts were normalized by trimmed mean of M-value method, and significance was determined *via* a generalized linear model quasi-likelihood F test using EdgeR package, reporting *p*-value, Benjamini–Hochberg multi-test corrected *p*-value, and *q*-value. Prior to GO and Kyoto Encyclopedia of Genes and Genomes (KEGG) enrichment analyses, the list of significant differentially expressed genes (*q*-value < 0.05) was split by logFC to produce 2 separate treatment-specific groups of data (solely related to 1—cold or 2—RT only). Data were analyzed and networks generated through the use of QIAGEN Ingenuity Pathway Analysis (21). Ingenuity pathway analysis (IPA) was performed from count, logFC, and *q*-value from the unfiltered, differentially expressed dataset. For IPA analyses, a 5% false discovery rate (FDR) threshold was applied to differentially expressed (DE genes), and a Z-score (-2.0 ≤ *Z* ≥ 2.0) was considered significant.

### Histology

#### Whole-Mount Adipose Immunostaining and Microscopy

The processing of scWAT for whole-mount immunofluorescent staining was performed as previously described (21). The primary antibodies used for immunofluorescent staining included the following: anti-GFP conjugated to Alexa Fluor 488, 1:200 (Invitrogen catalog #A21311), anti-tyrosine hydroxylase (TH), 1:500 (Millipore catalog #ab152); anti-myelin protein zero (MPZ), 1:250 (Abcam catalog #31851), anti-Synaptic Vesicle Glycoprotein 2A (SV2), 1:250 (DSHB catalog #SV2), and anti-Neurofilament M (2H3), 1:500 (DSHB catalog #2H3). The secondary antibodies for whole-mount immunofluorescent staining included the following: goat anti-rabbit IgG (H + L) highly cross adsorbed Alexa Fluor Plus 594, 1:500 (Invitrogen catalog #A32740) or Alexa Fluor 594 (Invitrogen catalog #A11005), and goat anti-mouse IgG (H + L) Alexa Fluor 488, 1:500 (Invitrogen catalog #A11001). Confocal imaging was performed on either a Leica TCS SP8 DLS or Leica Stellaris microscope with HyD detectors and white light laser. The objectives used were as follows: HC PL APO ×10/0.40 CS2, HC PL APO CS2 ×20/0.75, WD 0.62 mm, dry; HC PL APO CS2 ×40/1.30 oil, and HC PL APO CS2 ×63/1.40, WD 0.14 mm, oil. The images were processed with LASX software and pseudo-colored. A 4-kernel median noise filter was applied where the entire depot was imaged (**Figure 4E**).

#### Image Quantification

Using Fiji open-source platform for biological image analysis, mean fluorescence intensity quantification was performed (**Figures 4G**, **5G**) on maximum-projection micrographs of whole-mount scWAT. The images were converted to 8-bit grayscale, and a ROI was selected, including the SiLN and surrounding area. Equal thresholding was applied to all images within a cohort, and the mean intensity was measured.

#### Thin-Section Immunostaining

Both axillary and inguinal scWAT were collected from adult 7-day cold-exposed (5°C) LysM-Cre-Rosa eYFP reporter mice. Whole depots were cut into 100-um-thick slices longitudinally (from anterior to posterior). The slices from each depot were fixed in 2% paraformaldehyde for 1 h and then rinsed—for 10 min with 1× phosphate-buffered saline (PBS) with 10 U/ml heparin—twice at 4°C. The tissues were incubated in blocking buffer [1× PBS/2.5% bovine serum albumin (BSA)/0.5–1% Triton] at 4°C overnight. The tissues were rinsed in rinse buffer and stained with True Black^®^. The tissue slices were stained with anti-Post Synaptic Density Protein 95 (PSD95, 1:1,000, Abcam catalog #18258) for 48 h. The tissues were washed 3 times (at 1 h each) and incubated with secondary antibody overnight (Goat anti-Rabbit IgG Alexa Fluor 647, 1:1,000, Invitrogen catalog #21244). The tissues were washed 3 times (at 1 h each) and incubated with a primary antibody conjugated to GFP (Abcam catalog #ab6662 1:1,000) for 72 h, washed 3 times (at 1h each), and incubated overnight with DAPI for nuclear staining (100 ng/ml, Sigma-Aldrich catalog #D9564). The tissues were washed 5 times in rinse buffer (at 1h each) and mounted. The sections underwent z-depth reduction, as previously described (21), prior to mounting. The tissues were imaged on a Leica TCS SP8 DLS at ×63 magnification.

#### Flow Cytometry

SVF was isolated from scWAT depots as described above. Flow cytometric analysis was performed as previously described (22). The cells were labeled with fixable viability dye (eFluor506 or eFluor780; eBioscience), blocked with anti-CD16/32 (clone 2.4G2), and stained with fluorochrome-conjugated antibodies specific for CD11b (clone M1/70), Ly6G (1A8), CD45 (30-F11), IL4ra (mIL4R-M1), CD14 (rmC5-3), CD115 (T38-320), and CD11c (HL3), which were all purchased from BD Pharmingen. The fluorochrome-conjugated antibodies specific for CD101 (polyclonal) were purchased from eBiosciences. For intracellular staining, the cells were fixed and permeabilized with BD cytofix and cytoperm solutions and then stained with fluorescent antibodies specific for arginase-1 (polyclonal; eBiosciences), iNOS, and CD206 (MR6F3; eBiosciences). Flow cytometry was performed with a FACS Symphony A3 cell analyzer (BD Biosciences). The cells were gated on forward and side scatter after double exclusion and analyzed on FlowJo v10 software.

#### Magnetic-Activated Cell Sorting

Magnetic-activated cell sorting (MACS) was performed as previously described (12). Briefly, SVF from bilateral whole inguinal adipose depots was isolated and resuspended in degassed buffer containing 0.5% BSA and 2 mM EDTA in 1× PBS, pH 7.2. The resuspended cells were sorted on the MidiMACS Quadro magnetic-activated cell separator system (Miltenyi Biotec, Bergisch Gladbach, Germany) according to the manufacturer’s instructions. The cells were stained with primary PE-conjugated antibody, CD11b-PE (catalog #130-098-087); a 1:10 antibody dilution per 10^7^ cells was used. The cells were incubated for 10 min at 4–8°C, washed, and centrifuged at 300*g* for 10 min. The cell pellet was resuspended in anti-PE microbeads (1:10 dilution, Anti-PE MultiSort Kit catalog #130-090-757), incubated at 4–8°C for 15 min, washed, and centrifuged at 300*g* for 10 min. The cells were resuspended in 500 µl of buffer and passed through LS columns of the MACS separator (the LS columns were prepped according to the manufacturer’s instructions). The cells were washed 3x, and flow through containing CD11b- was collected. LS columns were removed from the MACS separator and flushed with 5 ml of buffer to release the magnetically labeled cell fraction (CD11b+). MicroBeads were removed using MicroSort release reagent (provided in Anti-PE MultiSort Kit catalog #130-090-757). MicroBead free CD11b+ cell fraction was labeled for the secondary marker, anti-F4/80-APC (catalog #130-102-942), following the same procedure as described for the primary marker, except that Anti-APC MicroBeads (catalog #130-090-855) were used.

#### RNA Isolation and RT-qPCR

Whole tissue depots were lysed and homogenized using TRI Reagent (ZYMO Research, catalog #R2050-1-200) with 0.9–2.0-mm RNAase-Free Stainless Beads (Next Advance, catalog #SSB14B-RNA) and a Bullet Blender. RNA was isolated using Direct-zol™ RNA MiniPrep kit (Zymo Research, catalog #ZR2052). RNA yield was determined on a Nanodrop; cDNA was synthesized using a High-Capacity Synthesis Kit (Applied Biosystems, Foster City, CA, USA). Real-time quantitative (q)PCR was performed with SsoAdvanced Universal SYBR Green Supermix (Bio-Rad, catalog #1725271) on a CFX384 instrument (Bio-Rad, Hercules, CA, USA). Gene expression was normalized to housekeeper peptidylprolyl isomerase A for analysis. The primer sequences for genes of interest can be found in **Supplementary Table S4**. For the statistical analysis of RT-qPCR results normalized to the housekeeper gene, an unpaired Student’s *t*-test was used.

#### BDNF ELISA

Whole scWAT depot protein lysates were prepared for tissue BDNF ELISA. Tissues (snap-frozen upon harvest) were minced in Pierce™ IP Lysis buffer (ThermoFisher Scientific catalog #87787) and homogenized using a Bullet Blender (Next Advance, Averill Park, NY, USA). Mouse BDNF PicoKine™ ELISA Kit (Boster Biological Technology, Pleasanton, CA, USA; catalog #EK0309) was used as per the manufacturer’s instruction to determine the amount of BDNF present in adipose protein lysates.

#### Statistical Analysis

For all animal experiments, age- and weight-matched mice were randomized to treatment groups. All plots represent mean ± SEM. Statistical calculations were carried out in Excel or GraphPad Prism software (La Jolla, CA, USA), utilizing ANOVA or Student’s *t*-test as indications of significance (specified in the figure legends). The error bars are SEMs (for all figures, *p < 0.05, **p < 0.01, ***p < 0.001, and ****p < 0.0001).

#### Ethical Statement

All procedures and handling of animals were performed in accordance with the University of Maine’s and The Ohio State University’s Institutional Animal Care and Use Committee (IACUC) in compliance with the guidelines of the PHS Policy on Humane Care and Use of Laboratory Animals and Guide for the Care and Use of Laboratory Animals. This study was approved by the University of Maine’s IACUC, under protocol A2017-09-04, and The Ohio State University, under protocols 2020A00000085 and 2021A00000004.

## Results

### Coordinated Changes to Nerve-Related Gene Pathways in Adipose Neuroimmune Cells After Cold Stimulation

**Figure 1:**
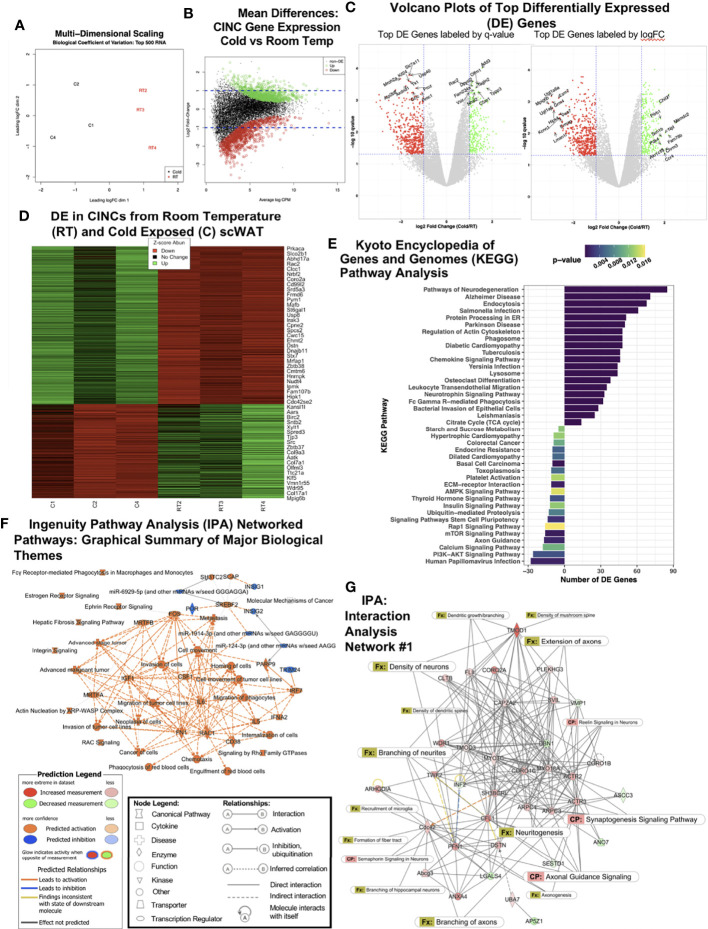
RNAseq of cold-induced neuroimmune cells (CINCs) from room temperature (RT) *versus* cold-stimulated scWAT. **(A)** Multidimensional scaling plot depicts expression differences and clustering in the top 500 transcripts in the cold (C1, C2, and C4 in black, left) and room temperature groups (RT2, RT3, and RT4 in red, right). **(B)** Visualization of the log-fold-change (logFC) difference between experimental groups *versus* the average expression across all samples (log count per million). Genes in green and red are differentially expressed following cold or room temperature treatment, respectively, while those in black are deemed not significant (*q*-value <0.05). **(C)** Volcano plots depict the transcriptomic profiles for cold-stimulated *versus* room temperature CINCs. Genes in green are upregulated, while genes in red are downregulated in cold-stimulated CINCs compared with room temperature CINCs and those in gray are not differentially expressed. The top 20 up- and downregulated genes are labeled by significance (*q*-value, left) or by expression (logFC, right). **(D)** Heat map showing the gene expression profile for the top up- and downregulated mRNA transcripts by Z-score. Z-score normalization was calculated across each gene (row of data). **(E)** Treatment-specific Kyoto Encyclopedia of Genes and Genomes (KEGG) analysis. Prior to KEGG database search, the differentially expressed gene list was filtered for the genes upregulated in cold (bars to the right) or room temperature (bars to the left). **(F)** Ingenuity pathway analysis (IPA) graphical summary of major biological themes. **(G)** IPA generated the top gene interaction network: cellular assembly and organization, cellular function and maintenance, tissue development. canonical pathways, and functions are overlaid to show the key molecules involved in neuronal pathways and nerve function. The IPA legends correspond to **Figures 1F, G**.

We previously demonstrated that CINCs (Ly6c+Ccr+Cx3cr1+ monocyte/macrophages) are recruited to inguinal scWAT upon cold exposure and that these cells expressed the neurotrophic factor BDNF and the beta3-adrenergic receptor, together indicating that they are neuroimmune cells capable of responding to noradrenergic stimulation (12). Considering these data and the prior findings by us and others that cold exposure leads to changes in adipose nerve density and patterning (3, 10), we aimed to further investigate the role that CINCs play in adipose tissue innervation. Adult (12–14 weeks old) male C57BL/6J mice were continuously cold-exposed at 5°C (cold) or maintained at room temperature (RT) in a diurnal incubator for 10 days. Following cold stimulation, SVF was isolated from inguinal scWAT *via* collagenase dissociation, and FACS sorting was performed to isolate Ly6c+Ccr+Cx3cr1+ cells. The RNA sequencing of 30 million single-end reads was performed on Ly6c+Ccr+Cx3cr1+ cells from room temperature and cold-exposed animals. Following hierarchal clustering (**Supplementary Figure S1A**), multi-dimensional scaling showed distinct and separate clustering of RT and cold CINCs (**Figure 1A**). Log fold change *versus* normalized mean counts (MA plot) showed the distribution of gene expression between cold and RT CINCs and revealed a substantial coordination (upregulation and downregulation) of genes in cold-exposed CINCs compared with room temperature CINCs (**Figure 1B**). The top 500 differentially expressed genes were then plotted by q-value (FDR < 0.05) against log fold change (logFC). The top 10 up- and downregulated genes labeled by *q*-value are shown in the left panel, while the top 10 up- and downregulated genes labeled by logFC are shown in the right panel (**Figure 1C**, **Supplementary Table S1**). Among the top upregulated genes in cold CINCs was dihydropyrimidinase-related protein 2 (*Dpysl2*) which is involved in neuronal development and polarity as well as axon guidance and growth cone collapse, axonal transport, and neurotransmitter release (23). *Dpysl2* encodes for collapsin-response-mediator protein 2 which is now known to be involved in neurite organization and remodeling in the central nervous system (24), although its role in the peripheral nervous system (PNS) remains understudied. A heat map of differentially expressed genes (**Figure 1D**) showed clear changes in gene expression between cold and RT CINCs, further indicating a coordinated and all-encompassing phenotypic and functional switch in these cells upon cold stimulation. The Gene Ontogeny (GO) analysis revealed changes in biological processes, cellular components, and molecular function of CINCs following cold exposure (**Supplementary Figures S1B–D**). Changes in biological processes included the upregulation of transport, metabolic, localization, and cellular processes in CINCS (**Supplementary Figure S1B**), while cell projection organization was most significantly downregulated in RT CINCs. Taken together, these gene expression changes are indicative of the plasticity of CINCs in response to cold stimulation.

Cold induced changes in CINCs were further investigated through the KEGG pathway analysis (**Figure 1E**). The KEGG pathway analysis revealed that CINCs have a clear gene expression pattern related to neuronal pathways, confirming that these are neuroimmune cells. “Pathways of neurodegeneration” contained the most upregulated DE genes in cold CINCs (**Figure 1E**). Specific genes within this pathway are shown in **Supplementary Table S2**. Upregulation of the neurotrophin signaling pathway was also observed, consistent with our previous findings that the neurotrophic factor BDNF is almost exclusively expressed in adipose SVF (3) and is required in the maintenance of adipose innervation (12). Chemokine signaling and leukocyte transendothelial migration pathways were also upregulated in cold CINCs (**Figure 1E**), consistent with our previous findings that these cells are recruited to adipose tissue with cold.

Compared to room temperature CINCs, cold-stimulated CINCs showed a downregulation of axon guidance pathway genes (**Figure 1E**), specifically those related to axon repulsion and growth cone retraction, including *Sema3*, *Sema4*, *Slit1*, *Efna2*, and *Efna3* (25) (**Supplementary Figure S1E**), suggesting that, in unstimulated conditions, CINCs play a role in axonal organization in adipose tissue, and with cold stimulation their neuroimmune function switches to promote axon outgrowth. Differential expression of neurotrophin signaling-related genes downstream of BDNF-TrkB signaling was also revealed, with cold-stimulated CINCs showing a higher expression of *Grb2 and Sos2* (**Supplementary Figure S1F**) compared with room temperature CINCs. The most significantly upregulated pathway in RT CINCs was the Rap1 signaling pathway (**Figure 1E**). Rap1 signaling is involved in various cellular processes, including cell adhesion and gap junction formation, cell migration, and cell polarization (26). Rap 1 signaling has been linked to a state of chronic inflammation and can induce a pro-inflammatory environment promoting inflammatory cytokine production *via* NFκB (27, 28). The starch and sucrose metabolism pathway was also strongly upregulated in RT CINCs compared with cold-stimulated CINCs, while the TCA cycle was upregulated in cold-stimulated CINCs (**Figure 1E**). Increases in glycolysis are associated with a proinflammatory phenotype (29, 30), which is consistent with the cell surface markers of CINCs. Stark changes in cellular metabolism accompany the phenotypic and functional switches in macrophages (31), and the DE between RT and cold-stimulated CINCs in these pathways further supports metabolic reprogramming in neuroimmune cells.

We used IPA to assess the coordinated changes in gene expression networks in cold-stimulated CINCs. The IPA-predicted pathways were in line with the pathways identified by KEGG, further supporting the neuroimmune role of CINCs (**Supplementary Figure S2A**). Among the activated pathways in cold-stimulated CINCs was noradrenaline and adrenaline degradation (**Supplementary Figure S2A**). Norepinephrine degradation has been reported as a key function of SAMs in adipose, which also express the Cx3cr1 marker and are recruited to adipose in obesity (17), and may represent a subset of adipose neuroimmune cell phenotypes. The major biological themes revealed by IPA are shown in **Figure 1F** and are represented as predicted canonical pathways, diseases, and functions that are either activated (orange) or inhibited (blue). This graphical summary revealed that more processes are activated rather than inhibited in cold-stimulated CINCs. It also revealed a central theme of cell movement related to the upregulation of colony stimulating factor-1 (CSF1), a potent monocyte/macrophage recruitment factor.

We looked at IPA networks ranked by the significance of molecules found together in a data set, which are ranked in the order of most to least significant. The top IPA network was shown to involve molecular functions related to cellular assembly and organization, cellular function and maintenance, and tissue development (**Figure 1G**). Canonical pathway and function annotations were overlaid to indicate the extent of nerve-related molecular interactions. The most significant function in Network 1 was revealed to be the branching of neurites (*p* = 0.000871 with 6 interacting molecules) (**Figure 1G** and **Supplementary Table S3**). The significance (*p*-values indicating the probability of finding this set of focus molecules in the network compared with a set of randomly selected molecules) for each function annotation for Network 1 can be found in **Supplementary Table S3**. Strikingly, in 9 out of the top 10 interaction networks, nerve-related functions including neuritogenesis, axonal guidance signaling, axon branching and extension, and synaptogenesis were predicted in cold-stimulated CINCs (**Figure 1G** and **Supplementary Figure S3**). A coordinated control of nerve plasticity-related genes in cold-stimulated CINCs was revealed when using IPA to examine singular functions predicted from our RNASeq dataset (**Supplementary Figure S2B**). Cold-stimulated CINCs were predicted to function to decrease neuronal cell death (*z*-score, -2.131; *p* = 1.00^-21^) and maintain neuritogenesis (*z*-score, 0.440; *p* = 1.61^-21^) (**Supplementary Figure S2B**).

### Validation of RNAseq Top Hits in Adipose Macrophages After Cold Stimulation

**Figure 2:**
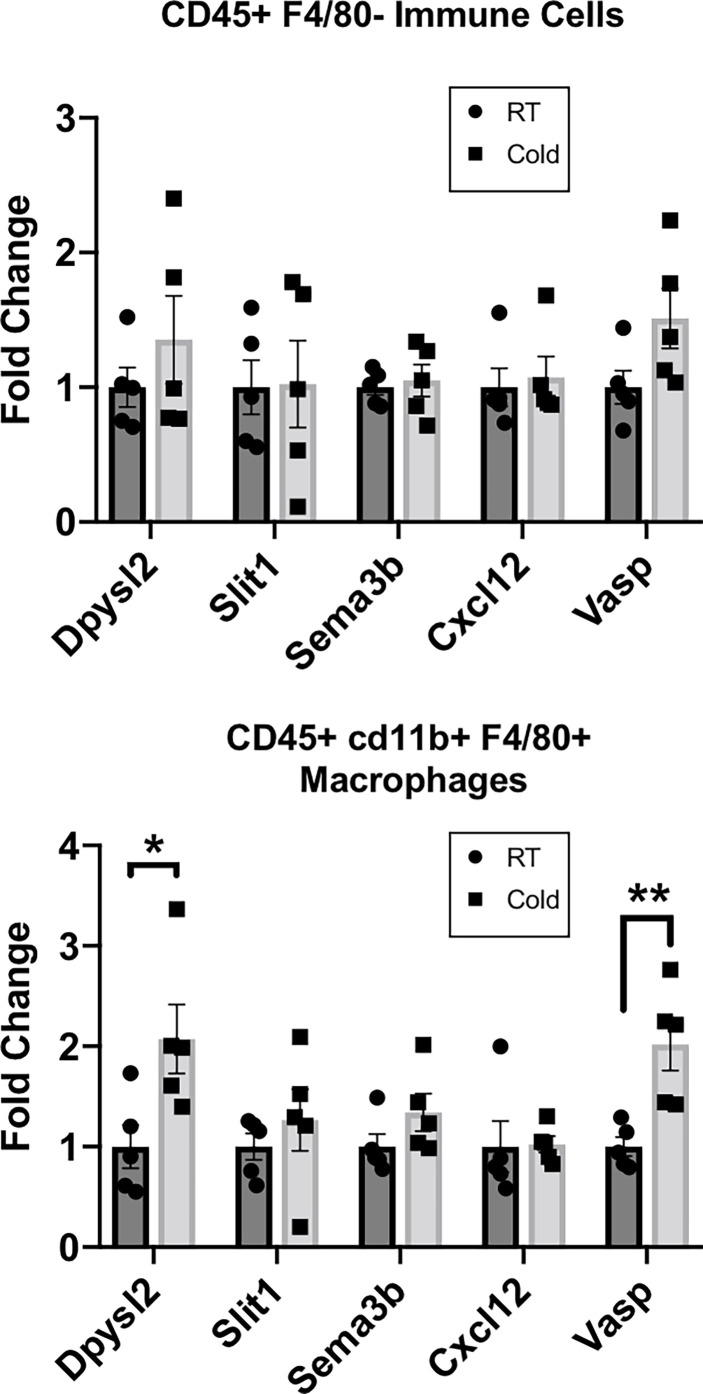
RT-qPCR of adipose stromal vascular fraction (SVF) cells following 5 days of cold exposure. Relative gene expression of select axonal guidance markers in myeloid immune cells (CD45+cd11b+/) and macrophage populations (CD45+ cd11b+F4/80+) sorted *via* magnetic-activated cell sorting from scWAT SVF of room temperature mice and cold-exposed (5°C) mice for 5 days. Adult (at least 12 weeks old) C57BL/6J male mice were used; *N* = 4 RT, *N* = 5 cold-exposed. Gene expression is shown as fold change in ΔΔCt values, normalized to room temperature control mice. Error bars are shown as SEM. *p-value < 0.05; **p-value <0.01.

Our RNASeq analysis revealed that cold stimulation alters the neuroimmune function of CINCs in scWAT to promote the pathways of neurite remodeling in the tissue. Therefore, we sought to determine if these gene expression changes are widespread in the overall scWAT macrophage population. We used MACS to isolate CD45+cd11b+F4/80+ macrophages from the SVF of scWAT maintained at room temperature *versus* those from 5-day cold-stimulated adult C57BL/6J male mice. *Dpysl2*, which was one of the top 10 differentially expressed genes in RNASeq analysis and upregulated in cold-stimulated CINCs (**Figure 1C**; **Supplementary Table S1**) was significantly upregulated in cold-stimulated *versus* RT CD45+cd11b+F4/80+ macrophages, consistent with the effects of cold stimulation on CINCs (**Figure 1C** and **Supplementary Table S1**), but this was not observed in the broader immune cell population (marked by CD45+ alone; **Figure 2**). Vasodilator-stimulated phosphoprotein (*Vasp*), a member of the ENA-VASP protein family that plays a role in macrophage polarization, cell adhesion, and motility and in axonal growth cone formation, was also significantly increased in 5-day cold-stimulated CD45+cd11b+F4/80+ macrophages but not the negative control cell population (**Figure 2**). *Vasp* was also identified in our RNASeq data set. These data indicate that a broader range of macrophages may play a neuroimmune function in scWAT, which warrants further investigation.

### CINCs Are Recruited to Adipose Tissue and Enriched Near Adipose Nerves By 48 h of Cold Exposure

**Figure 3:**
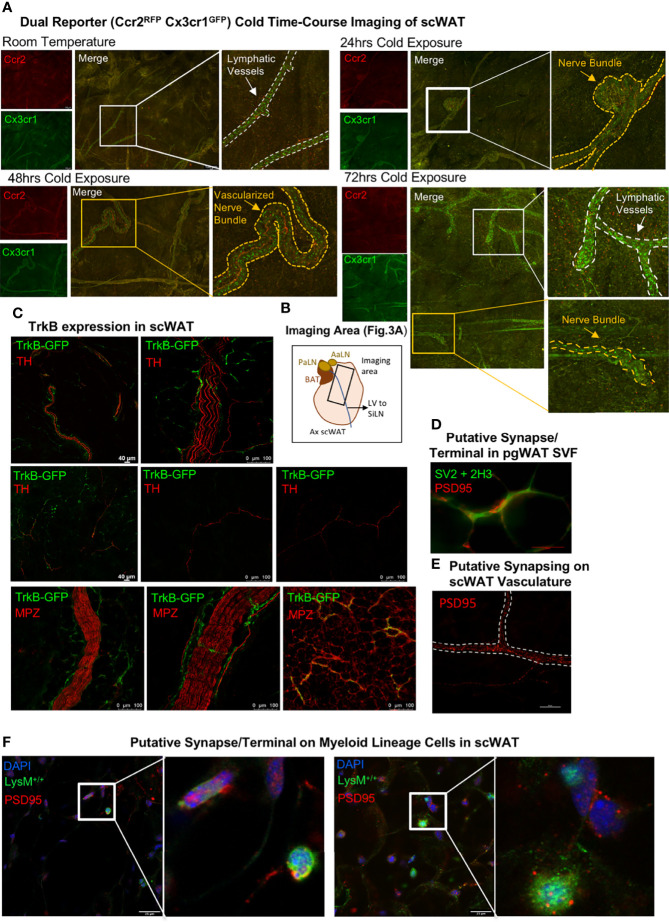
Whole-mount imaging of immune cell trafficking, TrkB expression, and identification of putative synapses in scWAT. **(A)** Adult (15–20 weeks old) male Ccr2^RFP^Cx3cr1^GFP^ dual-reporter mice were maintained at either room temperature (*N* = 2) or cold-exposed at 5°C for 24 h (*N* = 3), 48 h (*N* = 3), or 72 h (*N* = 3). scWAT was processed for whole -mount imaging as described in the method section. Tissue was imaged at ×10 on a confocal microscope equipped with white light laser. The images are maximum projections of merged tiled z-stacks. The lymphatic vasculature is outlined by a white dashed line, and the yellow dashed line outlines the nerve bundles. The images shown are representative of each group. **(B)** Schematic of the axillary scWAT imaged in (**A**). The black rectangle represents the area imaged. PaLN, proper axillary lymph node; AaLN, accessory axillary lymph node; BAT, brown adipose tissue; LV, lympthatic vessel; SiLN, subiliac lymph node. **(C)** Whole-mount imaging of TrkB expression in scWAT. scWAT from adult male Trkb^GFP^ reporter mice was immunolabeled with anti-GFP and either tyrosine hydroxylase or myelin protein zero and imaged by confocal microscopy at ×10 and ×20. **(D)** Perigonadal white adipose tissue from adult C57BL/6J mice was whole-mount processed and immunostained with markers for neurofilament and synaptic vesicles to identify the pre-synaptic axon terminal and the post-synaptic marker PSD95 (red). Imaged with ×40 objective lens on a Nikon E400 microscope. **(E)** Whole-mount imaging of scWAT immunostained with PSD95 revealed potential synapsing along a blood vessel (dashed white border). Imaged with ×40 objective lens using extended depth of field (NIS-Elements) on a Nikon E400 microscope. **(F)** Putative synapses on myeloid-lineage cells from LysM-Cre-Rosa YFP reporter mice. Tissues were co-stained with post-synaptic markers, PSD95 and DAPI, and imaged by confocal microscopy at ×63; z-max projections are shown. The inset is a digital zoom on PSD95 expressing myeloid-lineage cells.

Since CINCs appear to be recruited to adipose tissue upon cold exposure (12), we next aimed to determine the timing of their migration to and within scWAT by utilizing dual-reporter Ccr2^RFP^Cx3cr1^GFP^ mice. Adult (15–20 weeks old) male Ccr2^RFP^Cx3cr1^GFP^ mice were either maintained at RT, cold-exposed at 5°C for 24 h, cold-exposed at 5°C for 48 h, or cold-exposed at 5°C for 72 h. Whole-mount confocal imaging and collection of tiled z-stacks [as in (21)] revealed that, at RT, few Ccr2^RFP^Cx3cr1^GFP^ co-expressing cells are found within the adipose parenchyma, and only Cx3cr1^GFP^ cells were enriched within the lymphatic vasculature (**Figure 3A**, top left; **Figure 3B** indicates the imaging area for all groups). At 24 h of cold exposure, there was an accumulation of Ccr2^RFP^Cx3cr1^GFP^cells around adipose nerve bundles, with only some cells expressing Cx3cr1^GFP^ alone (**Figure 3A**, top right). By 48 h of cold exposure, more Cx3cr1^GFP^ cells and more Ccr2^RFP^ cells were observed within nerve bundles, with fewer Ccr2^RFP^Cx3cr1^GFP^ cells (**Figure 3A**, bottom left). By 72 h, Cx3cr1^GFP^ cells are again enriched in lymphatic vessels (**Figure 3A**, bottom right) but also still present within nerve bundles (**Figure 3A**, bottom right). The morphological changes in Cx3cr1^GFP^ cells found within nerve bundles were initially observed between 24 and 48 h of cold exposure (**Figure 3A** and **Supplementary Figure S4A**), suggesting a functional change during that time window. Specifically, at the 24-h post-cold timepoint, there were fewer elongated Cx3cr1^GFP^ cells within nerve bundles (**Supplementary Figure S4A**, top right panel), but by 48h the majority of Cx3cr1^GFP^ cells exhibited a distinct elongated shape and extended filipodia (**Supplementary Figure S4A**, bottom left panel), which is consistent with macrophage activation and polarization to a regenerative phenotype (32). This elongated morphology of Cx3cr1^GFP^ cells persisted within nerve bundles at 72 h of cold exposure and was also the predominant morphology of Cx3cr1^GFP^ cells observed within scWAT lymphatics (**Figure 3A** and **Supplementary Figure S4A**, bottom right panel).

### Whole-Mount Adipose Tissue Confocal Imaging Revealed TrkB Expression on Sympathetic Nerve Fibers and Identified Putative Synapses on SVF Cells

To determine the extent and localization of the neurotrophin receptor TrkB (which has highest binding affinity for BDNF and Ntf4/5) in scWAT, we used a TrkB^GFP^ reporter mouse. Whole-mount imaging of scWAT form adult (at least 12 weeks old) male TrkB^GFP^ mice revealed that TrkB is expressed on sympathetic nerves, marked by TH, including large TH+ nerve bundles and some individual TH+ axons (**Figure 3C**—top and middle panel; **Supplementary Figure S4B**). TrkB^GFP^ expression was also observed on large myelinated nerve bundles and thinly myelinated nerve fibers in scWAT as evidenced by TrkB^GFP^ and MPZ co-staining (**Figure 3C**—bottom panel; **Supplementary Figure S4B**). Of note is that TrkB^GFP^ is structurally co-localized and not fluorescently co-localized with both TH and MPZ (**Supplementary Figure S4B**) given the way myelin wraps around axonal membranes. This distribution of TrkB indicates expression on both sensory and sympathetic fibers. As a control, we confirmed that TrkB^GFP^ expression was also evident in the T12 dorsal root ganglia that contain cell bodies for the sensory nerves that innervate the scWAT, under both room temperature and cold-stimulated conditions (**Supplementary Figure S4C**). These data support previous findings of a role for neurotrophin signaling in scWAT (12).

We had previously reported that CINCs expressed the beta3-adrenegic receptor, and it is well documented that bidirectional communication exists between immune cells and peripheral nerves (33, 34). Therefore, we investigated whether a neuroimmune “synapse” may exist in adipose similar to that observed recently in the inguinal scWAT lymph node (35). Putative synapses were identified in the SVF of perigonadal white adipose tissue (**Figure 3D**) as well as on blood vessels (**Figure 3E**) and on myeloid-lineage SVF cells in inguinal scWAT (**Figure 3F**) using the post-synaptic marker PSD95. These findings support a potential means of neuroimmune crosstalk in adipose, although a *bona fide* neurological synapse/terminal/junction on adipose SVF cells has yet to be identified and validated.

### LysMCre+/-::BDNF-/- (KO) Mice Display Reduced Neurite Branching and Density Following Cold Exposure

**Figure 4:**
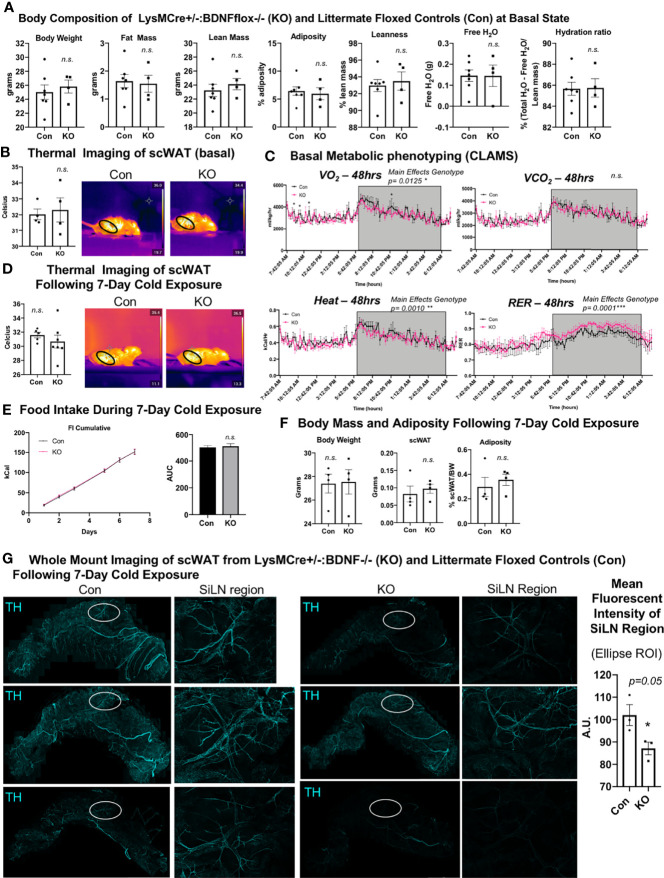
*LysMCre^+/-^::BDNF^-/-^* (KO) mice showed no difference in body composition, had a lower energy expenditure at the basal state, and exhibited decreased neurite density in scWAT upon cold exposure. **(A)** The body composition of adult (14 weeks old) male *LysMCre^+/-^:: BDNF^-/-^* (KO) and their littermate *LysMCre^-/-^::BDNF^fl/fl^* (Con) controls was measured by echoMRI; *N* = 7 Con, *N* = 4 KO. **(B)** The skin surface temperature above the inguinal scWAT of adult (12–14 weeks old) Con and KO mice at the basal state was measured using a thermal camera (FLIR T560); *N* = 4 Con, *N* = 4 KO. The mean temperature in the region of interest (indicated by the black circle on the thermal images) for *n* = 3 images per animal was qualified in FLIR Research Studio Application software, plotted in GraphPad Prism software, and analyzed by Student’s *t*-test (two-tailed). **(C)** Adult (16 weeks old) male Con (*N* = 3) and KO (*N* = 3) mice were assessed in metabolic cages (CLAMS) at the basal state. The KO mice displayed a lower oxygen consumption (VO_2_, top left panel); decreased energy expenditure is represented as heat calculated from the measures of VO_2_ and VCO_2_ (bottom left panel), and there was a higher respiratory exchange ratio (bottom right panel) compared with the littermate controls. Waveform analysis of the metabolic cage measurements taken at 15-min increments for 48 h and represented over a 24-h period. Two-way ANOVA was performed, and main effects were reported; *p* ≤ 0.0001. Time of day is indicated on the *x*-axis. The animals were maintained on a 12-h light/dark cycle (the gray box indicates the dark cycle). **(D–G)** Adult (19–21 weeks old) Con (*N* = 4) and KO (*N* = 4) male mice were cold-exposed at 5°C for 7 days. The mice were single-caged in a diurnal incubator (12-h light/dark cycle). **(D)** The skin surface temperature above the inguinal scWAT of adult (12–14 weeks old) Con and KO mice was measured at day 7 of cold exposure and analyzed as described in **(B)**. **(E)** Food intake was measured nearly daily throughout the cold exposure intervention. The area under the curve for cumulative food intake was calculated and compared for Con and KO animals. **(F)** The body mass and tissue weights were measured, and adiposity was calculated. Data were analyzed by Student’s *t*-test (two-tailed). **(G)** Whole-mount immunostaining and imaging were performed on scWAT from cold-exposed Con and KO male mice. The tissue was stained with tyrosine hydroxylase for the assessment of sympathetic innervation. Imaging was performed on a confocal microscope equipped with WLL using an objective at ×10 magnification. The images are z-maximum intensity projections (with median blur filter and 4 kernel size applied) of merged tiled z-stack imaging of the entire scWAT depot. White circles represent the SiLN region (inset to the right of each image) for which the mean intensity was quantified (far right). All error bars are SEMs. *p-value < 0.05; n. s., not significant.

We previously demonstrated that the knockout of BDNF from myeloid-lineage cells (LysM-Cre) resulted in reduced neurite density in scWAT (by western blot) and subsequent metabolic perturbations, including decreased basal energy expenditure, decreased induction of *Ucp1* in scWAT with cold exposure, and increased fat mass accumulation during a high-fat diet challenge (12). These findings reinforced the idea that adipose neuroimmune cells support the function and survival of peripheral nerves in the tissue. Here we again utilized the LysMCre-BDNF knockout model to further explore the role of myeloid-lineage cells in neurite plasticity in scWAT. Adult (14 weeks old) male mice in the basal state showed no difference in body composition as assessed by echoMRI (**Figure 4A**) and no difference in mean regional body temperature as determined by *in vivo* thermal imaging of skin surface temperature above inguinal scWAT (**Figure 4B**). Confirming our prior findings from experiments conducted at a different institutional mouse facility, basal physiological phenotyping in metabolic cages showed that KO mice had reduced energy expenditure compared with Con mice, especially at the start of the dark cycle when the mice eat more food as evidenced by a significant decrease in oxygen consumption (VO_2_) and heat production (**Figure 4C**, main effect of genotype reported). The RER indicated a higher reliance on carbohydrates for fuel, as opposed to lipids, for the KO animals (**Figure 4C**, main effect of genotype reported). Following 7 days of continuous cold exposure (in a diurnal chamber), the KO mice continued to exhibit no difference in skin surface temperature above inguinal scWAT compared with Con mice as determined by *in vivo* thermal (**Figure 4D**). There was also no difference in food intake during the 7-day cold exposure between KO and Con animals, supporting our previous findings that this model does not exhibit a central nervous system (CNS) phenotype (**Figure 4E**). There was also no difference in body weight, scWAT weight, or adiposity between Con and KO animals following cold exposure (**Figure 4F**). These data together are consistent with our previously reported findings (12).

We and others (3, 10) have reported cold-induced remodeling/repatterning of neurites in scWAT, which is particularly evident around the scWAT SiLN. To evaluate if myeloid cell-derived BDNF affects neurite branching and remodeling, we cold-exposed adult (19–21 weeks old) male KO and littermate Con mice for 7 days. We observed no change in body weight or adiposity in KO *versus* Con mice (**Figure 4F**) and a trend for decreased BDNF expression in whole scWAT (**Supplementary Figure S5A**). Whole-mount imaging of sympathetic innervation (TH staining) in scWAT was performed and revealed a marked decrease of neurite density/branching in KO animals compared with Con (**Figure 4G**). The most noticeable difference in neurite density/branching could be found around the SiLN region (**Figure 4G**, inset). The SiLN region exhibits the most plasticity in neurite density and remodeling in response to cold stimulation (3) and is also the area of scWAT with enriched cold-induced browning (36).

### Cx3cr1CreER+/-::BDNF-/- (KO) Mice Exhibit the Opposite Energy Expenditure Phenotype of LysMCre^+/-^::BDNF^-/-^ (KO) Mice

**Figure 5:**
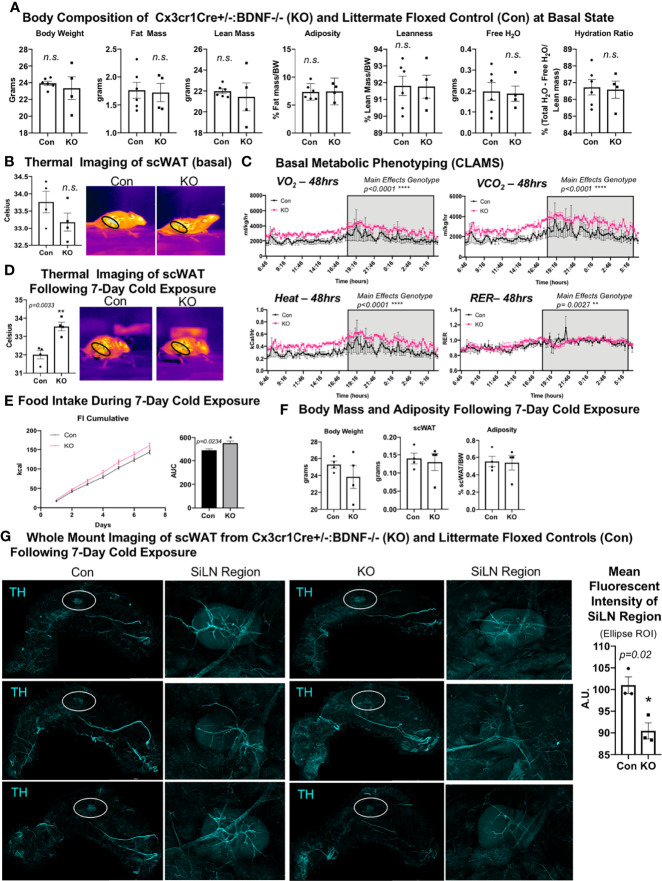
*Cx3crCreER1^+/-^::BDNF^-/-^* (KO) mice showed no difference in body composition and had a lower energy expenditure at the basal state and decreased neurite density in scWAT upon cold exposure. **(A)** The body composition of adult (14–16 weeks old) male *Cx3cr1CreER^+/-^:: BDNF^-/-^* (KO) and their littermate *Cx3cr1CrERe^-/-^::BDNF^fl/fl^* (Con) controls was measured by echoMRI; *N* = 6 Con, *N* = 4 KO. **(B)** The skin surface temperature above the inguinal scWAT of adult (14 weeks old) Con and KO mice at the basal state was measured using a thermal camera (FLIR T560); *N* = 4 Con, *N* = 4 KO. The mean temperature in the region of interest (indicated by the black circle on the thermal images) for *n* = 3 images per animal was qualified in FLIR Research Studio Application software, plotted in GraphPad Prism software, and analyzed by Student’s *t*-test (two-tailed). **(C)** Adult (25–27 weeks old) male Con (*N* = 3) and KO (*N* = 3) mice were assessed in metabolic cages (CLAMS) at the basal state. The KO mice displayed higher oxygen consumption (VO_2_, top left panel) and carbon dioxide respiration (VCO_2,_ top right panel); greater energy expenditure was represented as heat calculated from measures of VO_2_ and VCO_2_ (bottom left panel) and had a significantly altered respiratory exchange ratio (bottom right panel) compared with Con animals. Waveform analysis of metabolic cage measurements taken at 15-min increments for 48 h and represented over a 24-h period. Two-way ANOVA was performed, and the main effects were reported; *p* ≤ 0.0001. Time of day is indicated on the x-axis. The animals were maintained on a 12-h light/dark cycle (the gray box indicates the dark cycle). **(D)** Adult (14–16 weeks old) Con (*N* = 4) and KO (*N* = 4) male mice were cold-exposed at 5°C for 7 days. The skin surface temperature above the inguinal scWAT of adult (14–16 weeks old) Con and KO mice was measured at day 7 of cold exposure and analyzed as described in **(B)**. **(E)** Adult (14–16 weeks old) Con (*N* = 4) and KO (*N* = 4) male mice were cold-exposed at 5°C for 7 days. Food intake was measured daily throughout the cold exposure intervention. The area under the curve for cumulative food intake was calculated and compared for Con and KO animals. **(F)** The body mass and tissue weights were measured, and adiposity was calculated for cold-exposed Con (*N* = 4) and KO (*N* = 4) mice. Data were analyzed by Student’s *t*-test (two-tailed). All cold-exposed mice were single-caged in a diurnal incubator (12-h light/dark cycle). **(G)** Whole-mount immunostaining and imaging were performed on scWAT from cold-exposed Con and KO male mice. Tissue was stained with tyrosine hydroxylase for the assessment of sympathetic innervation. Imaging was performed on a confocal microscope equipped with WLL using an objective at ×10 magnification. Images are z-maximum intensity projections (with median blur filter and 4 kernel size applied) of merged tiled z-stack imaging of the entire scWAT depot. White circles represent the SiLN region (inset to the right of each image) for which the mean intensity was quantified. All error bars are SEMs. *p-value < 0.05; **p-value < 0.01, ****p-value < 0.0001; n.s., not significant.

We next investigated the effects of eliminating BDNF from Cx3cr1 cells, a subset of LysM myeloid-lineage cells and a marker of CINCs, using a tamoxifen-inducible Cx3cr1creER^+/-^::BDNF-/- (KO) model. Adult male (14–16 weeks old) mice were evaluated for body mass composition, which revealed no difference between KO and littermate Cx3cr1creER ^-/-^::BDNF^fl/fl^ controls (Con) (**Figure 5A**), similar to what was observed in the LysMCre KOs. Thermal imaging of skin surface temperature above the scWAT showed no difference in mean temperature of the inguinal scWAT region at the basal state between Con and KO mice (**Figure 5B**). However, CLAMS metabolic cage assessments performed at room temperature revealed increased energy expenditure (VO_2_, VCO_2_, and heat; **Figure 5C**) in the KO mice, and following 7 days of cold exposure, the KO mice exhibited a significant increase in scWAT skin surface temperature compared with the Con mice (**Figure 5D**). This was concomitant with increased food intake by KO animals during the 7-day cold exposure (**Figure 5E**) and no change in body weight or adiposity compared with Con mice (**Figure 5F**). The increased energy expenditure during CLAMS metabolic cage assessments (VO_2_, VCO_2_, and heat; **Figure 5C**) likely explains the compensatory increase in food intake and resulting maintenance of body weight and adiposity. Since Cx3Cr1-Cre may impact microglia in the brain (unlike LysM-Cre) and food intake can be driven by changes in hypothalamic neuropeptide expression, we assessed the hypothalamic gene expression and found no difference in *Bdnf* expression or in the expression of anorexigenic (*Pomc* and *Cart*) or orexigenic (*Npy* and *AgRP)* neuropeptides in the hypothalamus of KO *versus* Con animals (**Supplementary Figure S6A**). The gene expression in scWAT following cold exposure in this mouse line revealed a significant decrease in *Gap43* expression in the KO animals (**Supplementary Figure S6B**), suggesting that there may be changes in axonal growth cone formation or Schwann cell function in these mice. Taken together, Cx3cr1creER^+/-^::BDNF^-/-^ (KO) animals displayed an opposite metabolic phenotype from LysMcre^+/-^::BDNF^-/-^ (KO) animals, and this may have been due to compensatory effects, the adult induction of the Cre line by tamoxifen, or impacts on brain microglia. Furthermore, the LysMcre KO model resulted in the deletion of BDNF in multiple myeloid cells, likely resulting in broader adipose denervation.

**Figure 6:**
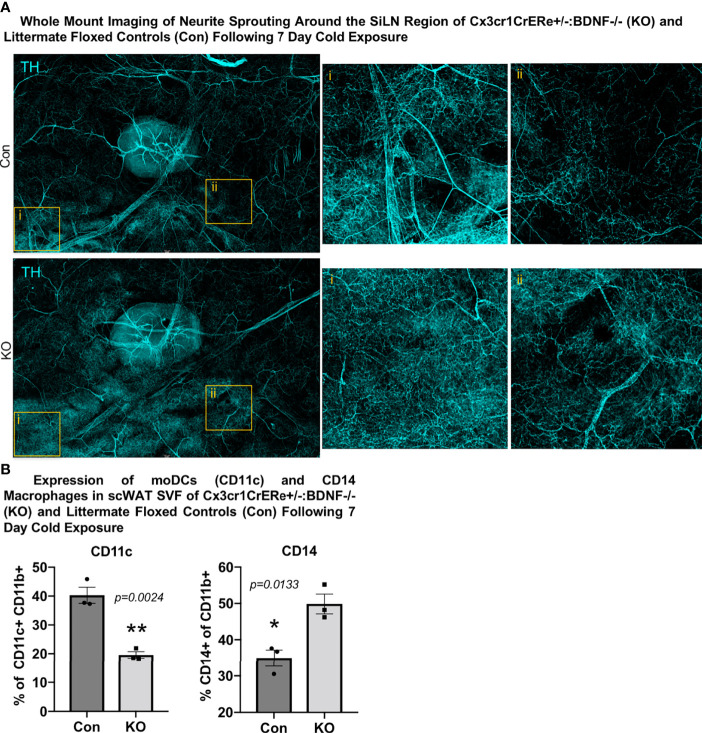
*Cx3crCreER1^+/-^::BDNF^-/-^* (KO) mice exhibited increased neurite density around the SiLN, decreased expression of CD11c+ adipose SVF cells, and increased expression of CD14+ adipose SVF cells compared with littermate floxed controls (Con). **(A)** Regional imaging (around SiLN) of whole-mount scWAT depots described in **Figure 5G**, where neurite sprouting was most pronounced. Images are representative of *N* = 3 per group. **(B)** Adult (15–17 weeks old) male *Cx3cr1CrERe^+/-^:BDNF^-/-^* (KO) and their littermate *Cx3cr1CrERe^-/-^::BDNF^fl/fl^* (Con) mice were cold-exposed (5°C) for 7 days. Stromal vascular fraction from bilateral inguinal scWAT depots was isolated, and flow cytometry was performed. Percentage change of CD11b+ CD11c+ cells and CD11b+ CD14+ cells in Con and KO mice. Data were analyzed by two-tailed Student’s *t*-test; *N* = 3 per group. All error bars are SEMs. *p < 0.05, **p < 0.01.

Surprisingly, despite the increased energy expenditure of Cx3cr1creER^+/-^::BDNF^-/-^ KO mice compared with Con (**Figure 5C**), whole-mount imaging of sympathetic innervation (TH staining) in their scWAT revealed a significantly decreased neurite density/branching around the SiLN region in KO animals (**Figure 5G**). The changes in patterns of innervation between Con and KO animals were most evident by the decrease in large nerve fibers within the SiLN region of KO animals (**Figure 5G**, insets). The loss of large fibers in KO animals within this area appears to be counteracted by a pronounced increase in small fiber branching/sprouting (**Figure 6A**). In the hundreds of scWAT depots we have imaged over the past several years, we have never seen small fiber density and branching in adipose to this extent.

Considering these unexpected findings, we assessed—by unbiased flow cytometry—the myeloid-derived immune cell populations in the inguinal scWAT of Con and KO animals following 7 days of cold exposure (**Supplementary Figures S6C–E**). The t-distributed stochastic neighbor embedding analysis revealed differences in immune cell population distribution between Con and KO animals (**Supplementary Figure S6D**). Most notably, KO animals exhibited a significant decrease in CD11c+ tissue-resident monocyte-derived dendritic cells (moDCs) and an increase in infiltrating CD14+ macrophages (**Figure 6B**, **Supplementary Figure S6E**). CD14-expressing myeloid-lineage cells have been shown to be neuroprotective in the CNS, where they also promoted axon regeneration *via* growth factor secretion (22). It is possible that the CD14+ macrophages seen here function in a similar manner. Whether the loss of moDCs in KO animals is compensated by the recruitment of CD14+ macrophages expressing the alternatively activated marker mannose receptor is unknown, although it is possible that the loss of BDNF from Cx3cr1+ has affected the development and function of other scWAT immune cell populations, leading to the differential expression of CD11c+ and CD14+ cells between the Con and KO animals as neurotrophins have been shown to have autocrine effects on immune cells (37). Taken together, it is clear that the loss of BDNF in Cx3cr1+ cells leads to changes within the adipose immune cell population, which may drive the observed reorganization of scWAT innervation (**Figure 6A**).

## Discussion

Taking our CINC RNAseq data and tissue imaging data together, we have demonstrated that adipose tissue neuroimmune cells are closely associated with tissue axons and nerve bundles, and in response to cold stimulation, these monocyte/macrophages turn on a coordinated gene expression program that indicates that they are supporting nerve survival and plasticity. Based on investigations of other similar myeloid-lineage immune cells in the body, the CINC markers (Ly6c/Ccr2/Cx3cr1) indicate that these cells are in a transient state (co-expressing Ccr2 and Cx3cr1), are recruited to the tissue (Ly6c and Ccr2 markers), are pro-inflammatory/regenerative (Ly6c, Ccr2, and Cx3cr1 markers), and are classical neuroimmune cells (Cx3cr1 marker). In addition, these cells exist in low numbers in the tissue (3,000–5,000 cells per pooled inguinal depots on average) and do not appear to adhere to the typical M1/M2 adipose tissue macrophage paradigm, and using at least 2 of the markers (Ccr2^RFP^ Cx3cr1^GFP^ dual-reporter mice), we found that these cells appear to move into and/or out of adipose tissue *via* the tissue lymphatic system and then associate with the adipose nerves in a transient and acute period of 0–72 h of cold stimulation [**Figure 3A** and (12)].

Our RNAseq data revealed a coordinated phenotypic switch of CINCs in response to cold, including changes to their cellular metabolism, consistent with entering a state of activation, alterations in phagocytic activity, and other non-neuroimmune related functions. This revealed that cold-stimulation of skin thermal receptors promotes sensory nerve communication to the brain, resulting in sympathetic drive to adipose tissue which recruits monocyte/macrophages to the tissue and also drives the phenotypic switching of these cells, potentially *via* norepinephrine signaling. Together with the expression of adrenergic receptors on CINCs and the close association of adipose nerve endings forming a putative synapse with SVF neuroimmune cells, our data indicate that there is likely neuronal control over this coordinated phenotypic switch in gene expression networks in response to cold. These myeloid cell phenotypic changes may also be beneficial for maintaining metabolic health beyond adipose innervation remodeling—for example, Fc-gamma receptor (FcγR)-dependent phagocytosis, which we see upregulated in cold-stimulated CINCs (**Figures 1E, F**), is impaired in monocytes of type II diabetes patients with chronic hyperglycemia (38).

In many peripheral tissues, ranging from lung and intestine to skin and adipose tissue, macrophages have been found to be closely associated with the PNS (39). Adipose tissue neuroimmune cells have been described as nerve-associated macrophages (40), SAMs (17), and, as we have identified, CINCs (12). Neuroimmune cells, such as these monocyte-derived macrophages, can fulfill numerous roles from degrading norepinephrine in obese adipose in the case of SAMs to supporting neurite survival and outgrowth in the case of CINCs and other myeloid-lineage neuroimmune cells. Numerous studies investigating neural plasticity have found that inflammatory immune cells play an important, yet transient, role in nerve injury, regenerating nerves and promoting neurite outgrowth (22, 41). However, neuroimmune cells show a high heterogeneity in the PNS and are not restricted to a single phenotype (42). Macrophages involved in nerve injury and repair show a complex phenotype, with temporal-based expression of pro-inflammatory and anti-inflammatory genes and expression of growth factors including neurotrophins across both phenotypes (43), demonstrating that nerve regeneration and remodeling relies on a dynamic immune cell response. Regenerative capacity is highest in animals such as fish, which have less immune competence (reviewed in (44)), highlighting the important interaction and tradeoff between immunity and tissue remodeling/plasticity.

Peripheral nerves are highly regenerative, and macrophages are critical for their repair (reviewed in (45)). Macrophages can promote angiogenesis, enhance migration and proliferation of Schwann cells, and phagocytose debris from neuronal injury. Monocytes often make the transition to macrophages once they are recruited to a tissue after an injury and then begin secreting factors that mediate vascularization and extracellular matrix remodeling, and they also produce growth factors—all of which are important for nerve repair. Macrophages also recruit T and B lymphocytes that are similarly important for nerve regeneration (46). In recent years, the concept of a neuroimmune unit has become increasingly relevant, given the importance of nerve–immune cell interactions for tissue plasticity and physiology in response to environmental changes [reviewed in (47)].

As one example in adipose, it is already known that SAMs, which increase with obesity (a state associated with adipose neuropathy), can internalize and degrade norepinephrine (17). Thus, the recruitment of nerve-nourishing macrophages, such as CINCs that carry BDNF, and potentially phenotypically switching SAMS away from a state that reduces sympathetic nerve activity may be beneficial for obese and diabetic adipose tissues. The recruitment of CINCs as well as potentially other yet-to-be-identified adipose tissue myeloid-lineage neuroimmune cells may be a transient event, with the tissue quickly resolving the pro-inflammatory phenotype to ensure that inflammation does not lead to chronic insulin resistance. In mice without the chemokine receptor Ccr2, indeed the recruitment of macrophages to sites of nerve injury was decreased, thus blunting nerve repair (reviewed (48)). Given the surface expression of Ccr2 and other chemokine/chemoattractant receptors (including Ccr4 and Cx3cr1) on CINCs and potentially other recruited neuroimmune cells honing to adipose, we predict that they are responding to the chemokines secreted after cold stimulation (such as Ccl2) in order to be recruited to scWAT after cold stimulation, thus allowing them to release growth factors, including BDNF, that promote neurite outgrowth in the activated tissue. Our new RNAseq data further support this working model, given the cold-stimulated upregulation of CSF1, DE of genes in leukocyte transendothelial migration pathway (**Figure 1E**), and multiple predicted functions related to cell motility, including “homing of cell”, “chemotaxis”, and “migration of phagocytes” (**Figure 1F**).

Even in humans undergoing metabolically beneficial fasting, the switch from glucose to lipid fuel utilization was accompanied by an enhanced inflammatory signature in adipose biopsies (49), again indicating, like in cold or exercise situations, that acute inflammation is likely metabolically beneficial by promoting tissue plasticity and regeneration. The fibrosis observed in adipose after chronic inflammation may be a result of improper tissue repair and regeneration, as is known to occur when remodeling cannot be carried out properly by immune cells or in an inflammatory state in the tissue (50–52). Furthermore, the inflammatory roles of macrophages can be counteracted by the autonomic nervous system, and this may be impaired in cases of tissue neuropathy, leading to chronic inflammation. Finally, lipids released by adipose lipolysis and/or carried to immune cells in the lymphatic vasculature may be “resolving” the proinflammatory state of neuroimmune cells, but this may be dysfunctional in obesity/diabetes when neuropathy occurs. Taken together, there is clearly an important neuroimmune interaction in peripheral tissues that allows for the mediation of neural plasticity, but this may needs to be carefully regulated in order to not develop a longer-term immune dysregulation, such as the adipose inflammation observed with obesity.

Beyond our previously identified role for CINCs to release BDNF in adipose tissue, RNAseq data after cold stimulation has revealed coordinated changes in gene expression networks that point to additional neuroimmune functions, such as (1) secretion of neurotrophic factors (Ntf4/5), (2) response to growth factors (*Trkb*, *Grb2*, and *Sos2*), (3) axonal guidance, synaptogenesis, and neurite branching and outgrowth (see the top network in IPA analysis in **Supplementary Table S3**), (4) pruning and degradation of axons (see the top KEGG pathway in **Supplementary Table S2** and **Figure 1E**) as would be required for remodeling of tissue innervation patterns, and (5) degradation of neurotransmitters (see the pathway implicated in **Supplementary Figure 1E**).

Since many of these neuroimmune functions could be regulated by neurotrophic factors, which are pleiotropic growth factors capable of supporting nerve health and plasticity *via* a wide variety of mechanisms, we revisited the impact of myeloid-lineage BDNF deletion on adipose tissue innervation and neuroimmune status using two Cre-Lox models: the LysM-Cre × BDNF-flox model [deletion in all myeloid-lineage cells, as we have reported previously (12)] and the Cx3cr1-CreER × BDNF-flox model (which would more specifically target CINCs and other macrophages expressing Cx3cr1, such as SAMs). Interestingly, both models exhibited decreased innervation specifically around the SiLN region (**Figures 4G**, **5G**) despite the increased energy expenditure phenotype in Cx3cr1-BDNF KO mice (**Figures 5C,D**). A possible explanation for these apparently conflicting data can be found in the changes to innervation patterns in the Cx3cr1-BDNF KO mice compared with their littermate floxed controls and the LysM KO tissues. The loss of scWAT innervation within the SiLN region in the Cx3cr1-BDNF KO model was restricted to larger nerve fibers and was concomitant with a high density of small fiber TH+ neurites (**Figure 6A**). Cold exposure has been shown to increase the density of thin TH+ parenchymal fibers, specifically in areas of high browning density (53). It is possible that this increase in small fiber TH+ neurites results in more than enough norepinephrine release, allowing for thermogenesis to occur under conditions of cold exposure. Furthermore, the LysM-Cre model contains a broad promoter with efficiency in targeting multiple myeloid cells, including macrophages (both resident and recruited), monocytes, dendritic cells, NK cells, eosinophils, and neutrophils. On the other hand, Cx3cr1-CreER targets predominantly infiltrating monocytes/macrophages, microglia, dendritic cells, and NK cells, and deletion efficiency appears to vary by tissue type (54, 55). The broader myeloid cell BDNF deletion in the LysM-Cre KO model may account for the worse metabolic phenotype in those mice. In the Cx3cr1-CreER KO model, the deletion of BDNF may lead to compensatory BDNF production by other tissue-resident or recruited immune cells (56) or Schwann cells (6, 57) since *Bdnf* was not significantly reduced in scWAT of Cx3cr1-CreER^-/+^::BDNF^-/-^ (KO) mice (**Supplementary Figure S6B**) unlike the significantly decreased *Bdnf* expression previously seen in the LysM-Cre KO model (12).

Additional caveats of these two models need to be considered, such as that LysM-Cre is consistently shown to have no (58) or less (59) targeting to brain microglia *versus* the Cx3cr1-CreER, which is very effective at targeting microglia (60). Cx3cr1 expressing microglia appear to be derived from the embryonic yolk sac and seed the brain earlier than other myeloid-lineage cells (61), and the fact that the Cx3cr1 model is tamoxifen-inducible, this allows adult-onset deletion of BDNF but also confounds metabolic studies due to the known metabolic activity of tamoxifen (62). Given our data in the Cx3cr1-BDNF KO mice that exhibit changes in appetite and likely increased sympathetic drive to scWAT, there is a potential that central pathways are impacted *via* microglia KO of BDNF; however, we found that hypothalamic BDNF and appetite neuropeptides were unchanged in KO mice [similar to what we observed previously in the LysM-BDNF KO model (12)]. In addition, the Cre-Lox approach, in general, may not be ideal for these studies, given the ability of monocyte/macrophages to exhibit such transient plasticity, the movement in and out of tissues in response to various stimuli, and the inability to target specific subsets of tissue-specific neuroimmune cells that co-express multiple markers. Regardless of these limitations, our data clearly indicate that there is a role for myeloid-lineage neuroimmune cell-derived BDNF in regulating whole-body energy balance. These data together provide new evidence for how the altered BDNF/TrkB observed in some obese humans may be predisposing them to dysregulated energy balance beyond impacts in the brain. In addition, given our new RNAseq data of cold-stimulated CINCs, we cannot rule out that additional neurotrophic factors, acting alone or in concert with BDNF, may also be contributing to the maintenance and/or plasticity of adipose innervation. However, the presence of TrkB on a subset of adipose tissue nerves does indicate the importance of BDNF.

## Data Availability Statement

The original contributions presented in the study are publicly available. This data can be found here: NCBI, PRJNA804888.

## Ethics Statement

This study was approved by the University of Maine’s Institutional Animal Care and Use Committee, under protocol A2017-09-04, and The Ohio State University, under protocols 2020A00000085 and 2021A00000004.

## Author Contributions

MB and KT conceptualized the studies, designed the experiments, and analyzed the data. MB carried out the experiments. MB and KT both oversaw the project. MG conducted the RNAseq analysis. GG conducted qPCR experiments. JW conducted the adipose immunofluorescent imaging. AS conducted the flow cytometry in the Cx3CR1 KO line. All authors contributed to the article and approved the submitted version.

## Conflict of Interest

The authors declare that the research was conducted in the absence of any commercial or financial relationships that could be construed as a potential conflict of interest.

## Publisher’s Note

All claims expressed in this article are solely those of the authors and do not necessarily represent those of their affiliated organizations, or those of the publisher, the editors and the reviewers. Any product that may be evaluated in this article, or claim that may be made by its manufacturer, is not guaranteed or endorsed by the publisher.
