# Supplementary figures and images for: Adipose Tissue Myeloid-Lineage Neuroimmune Cells Express Genes Important for Neural Plasticity and Regulate Adipose Innervation
Source: Front Endocrinol (Lausanne). 2022 Jun 20;13:864925. doi: 10.3389/fendo.2022.864925 (PMC9251313; doi:10.3389/fendo.2022.864925)

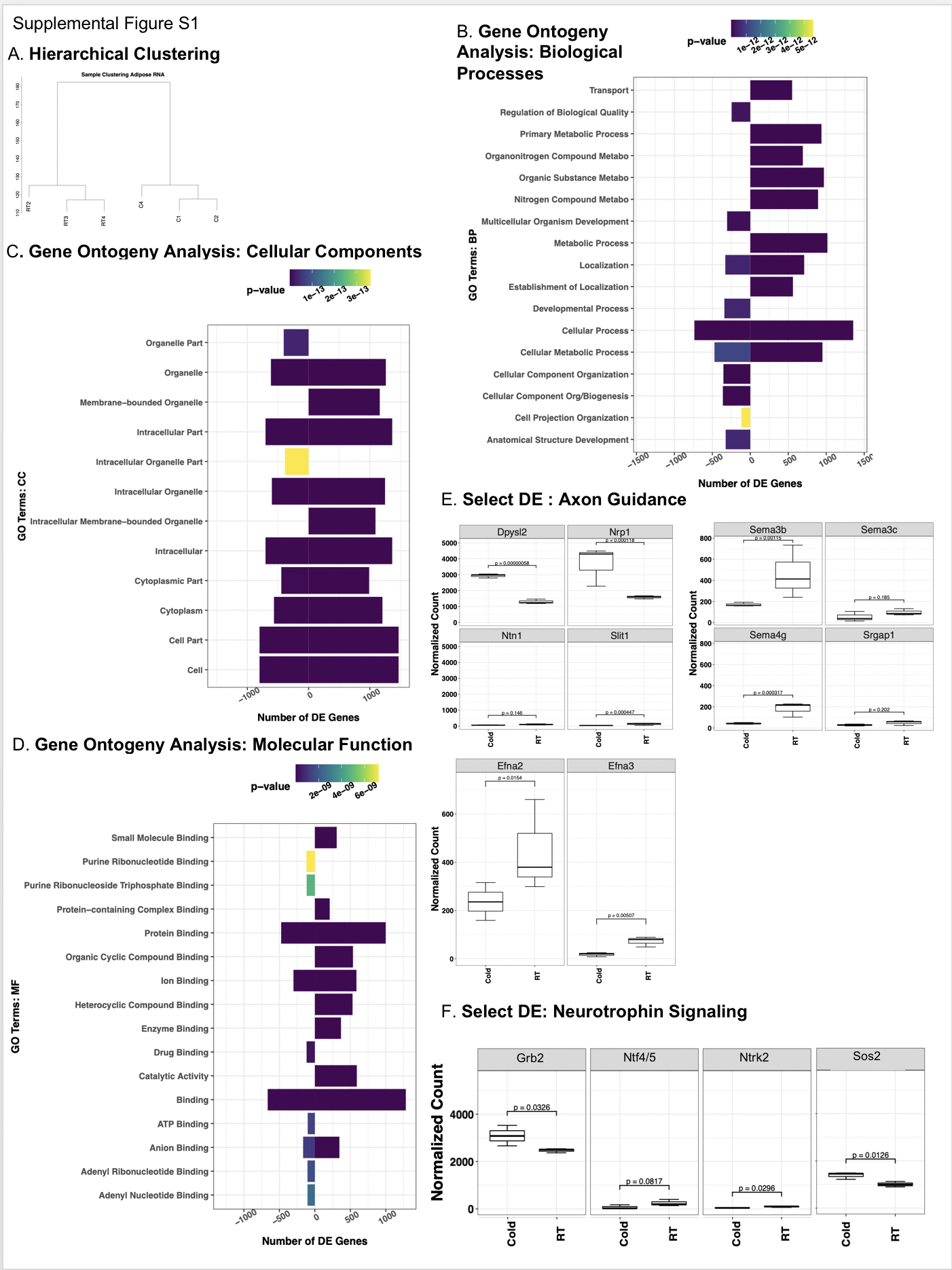

Supplement: Supplementary file 2 [file Image_1.tiff]

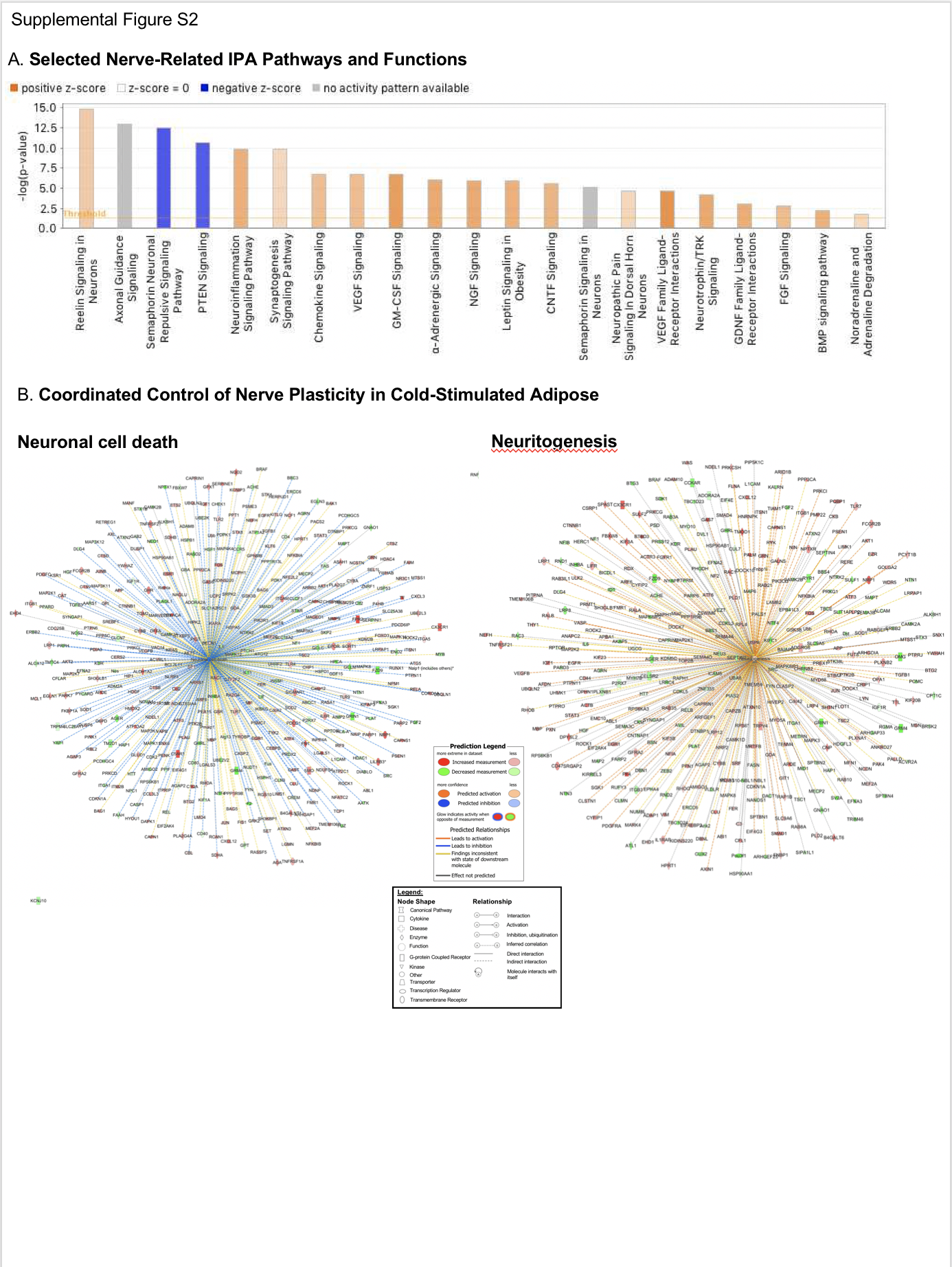

Supplement: Supplementary file 3 [file Image_2.tiff]

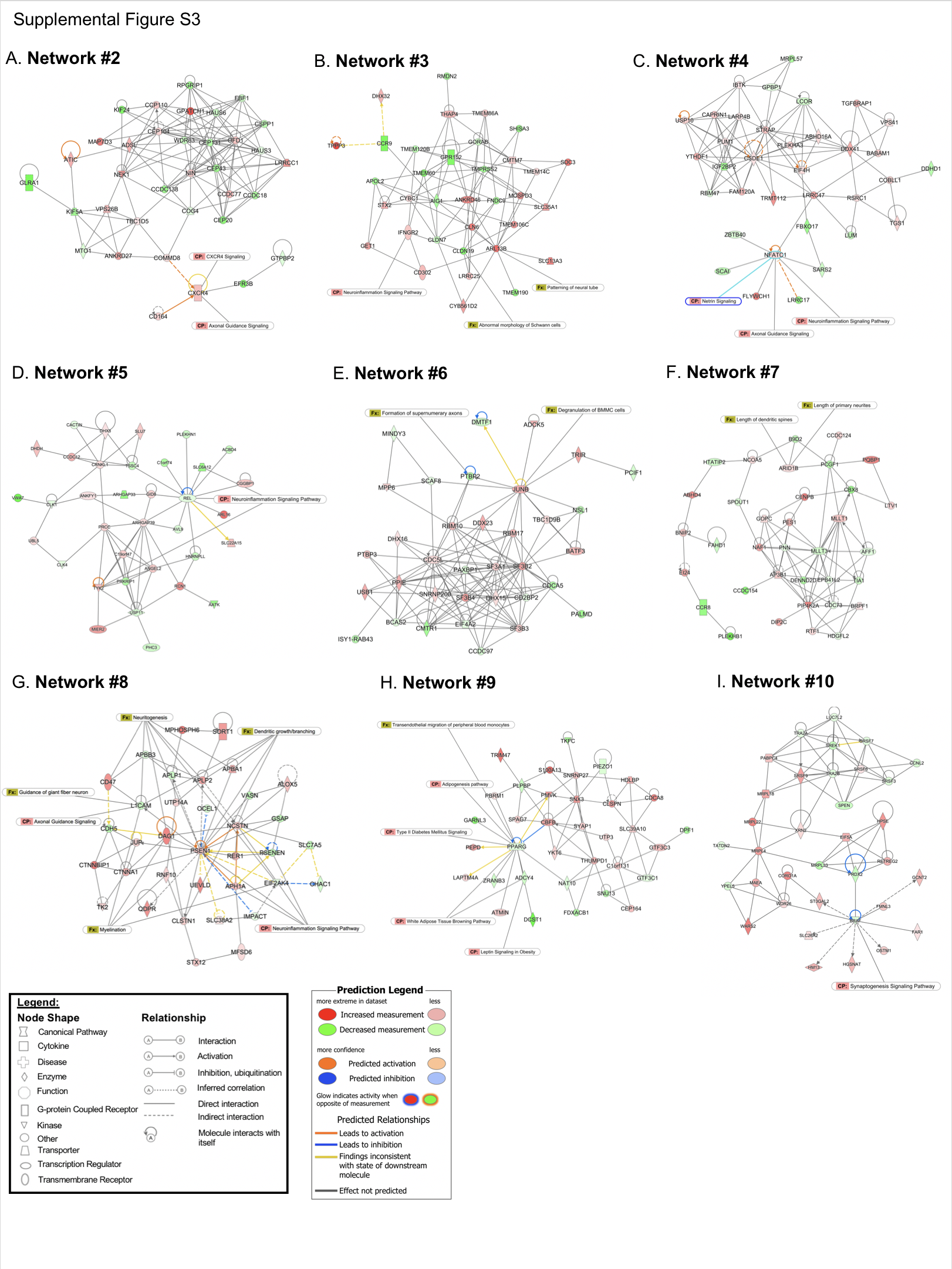

Supplement: Supplementary file 4 [file Image_3.tiff]

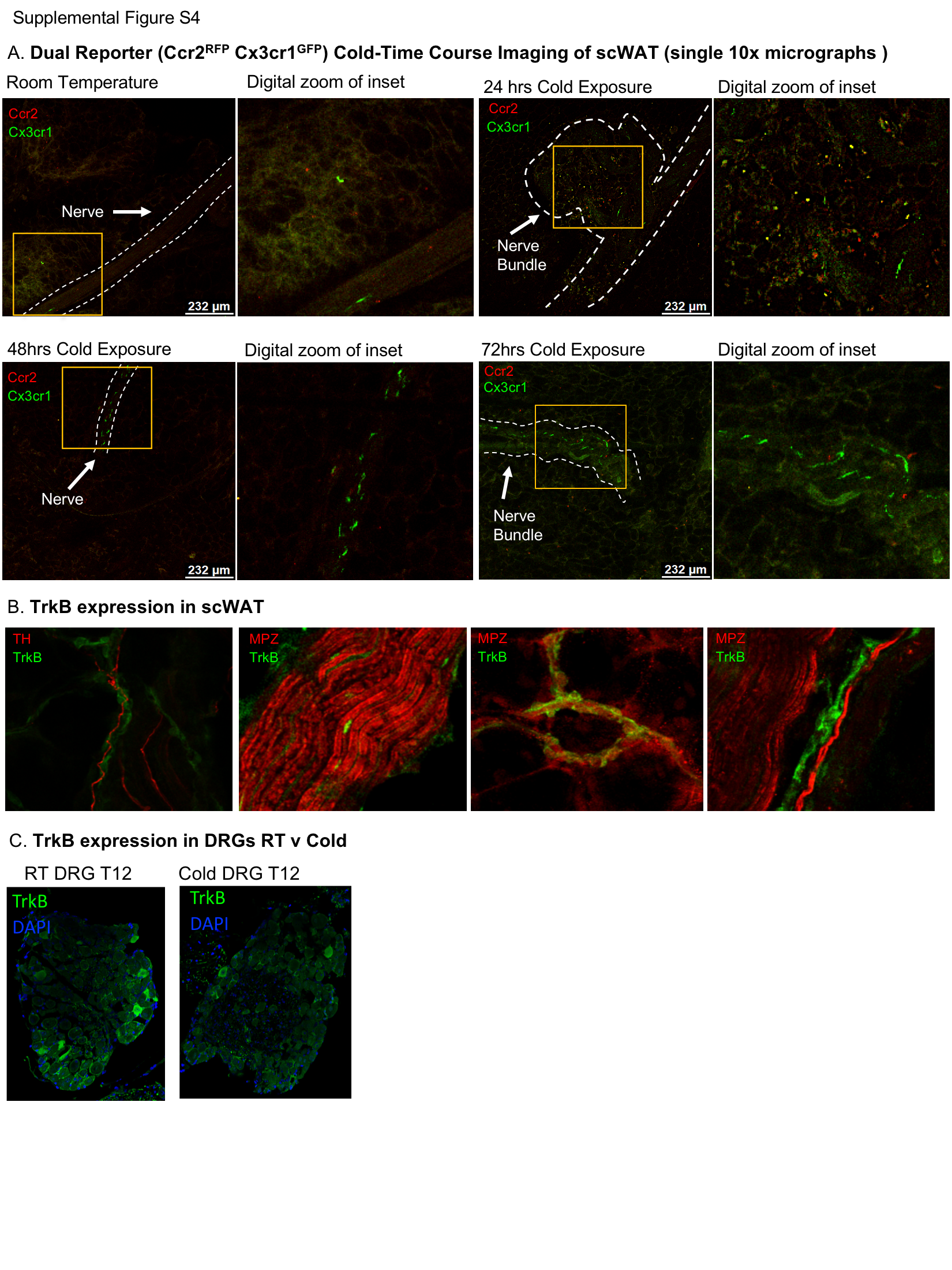

Supplement: Supplementary file 5 [file Image_4.tiff]

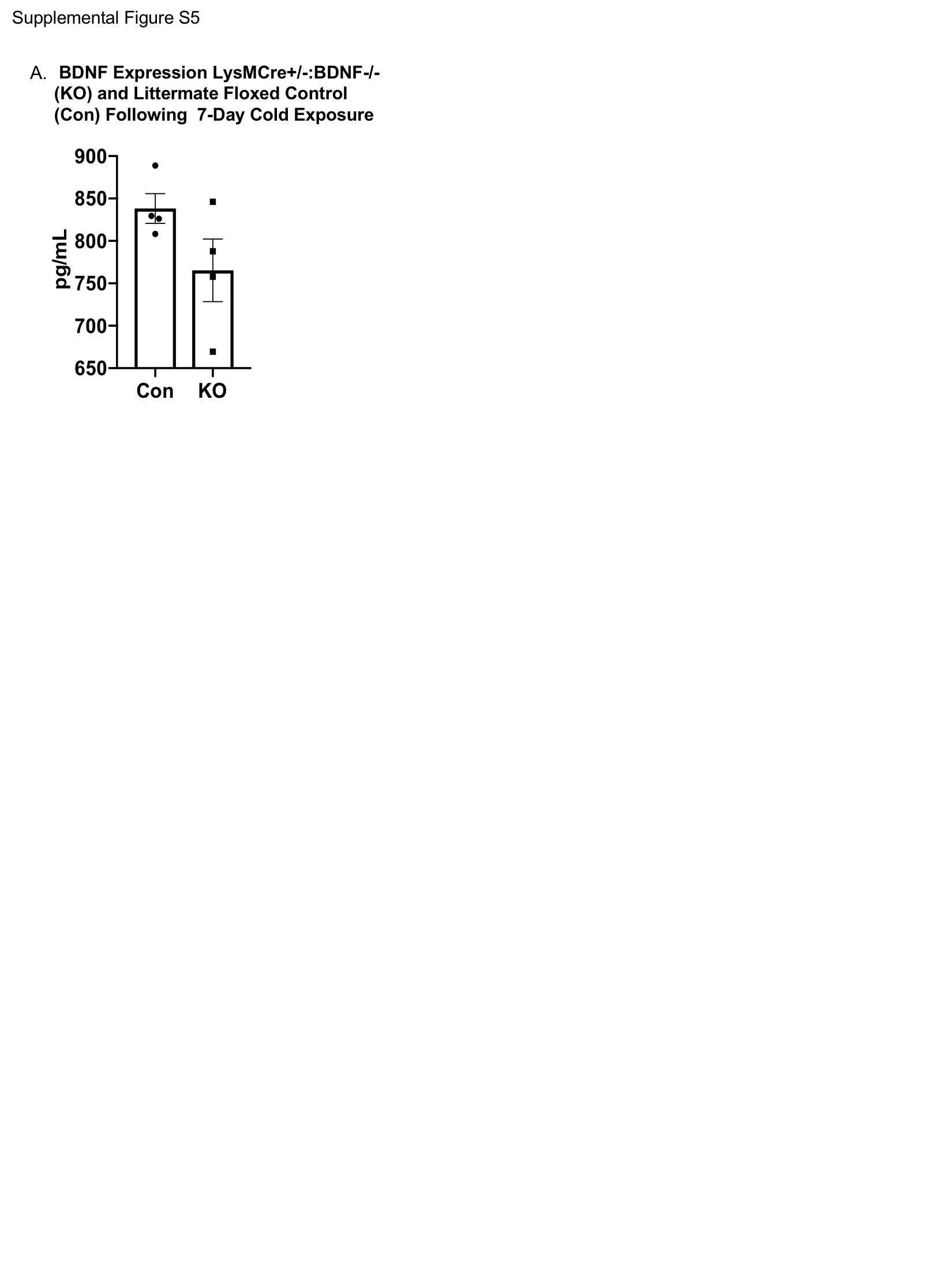

Supplement: Supplementary file 6 [file Image_5.tiff]

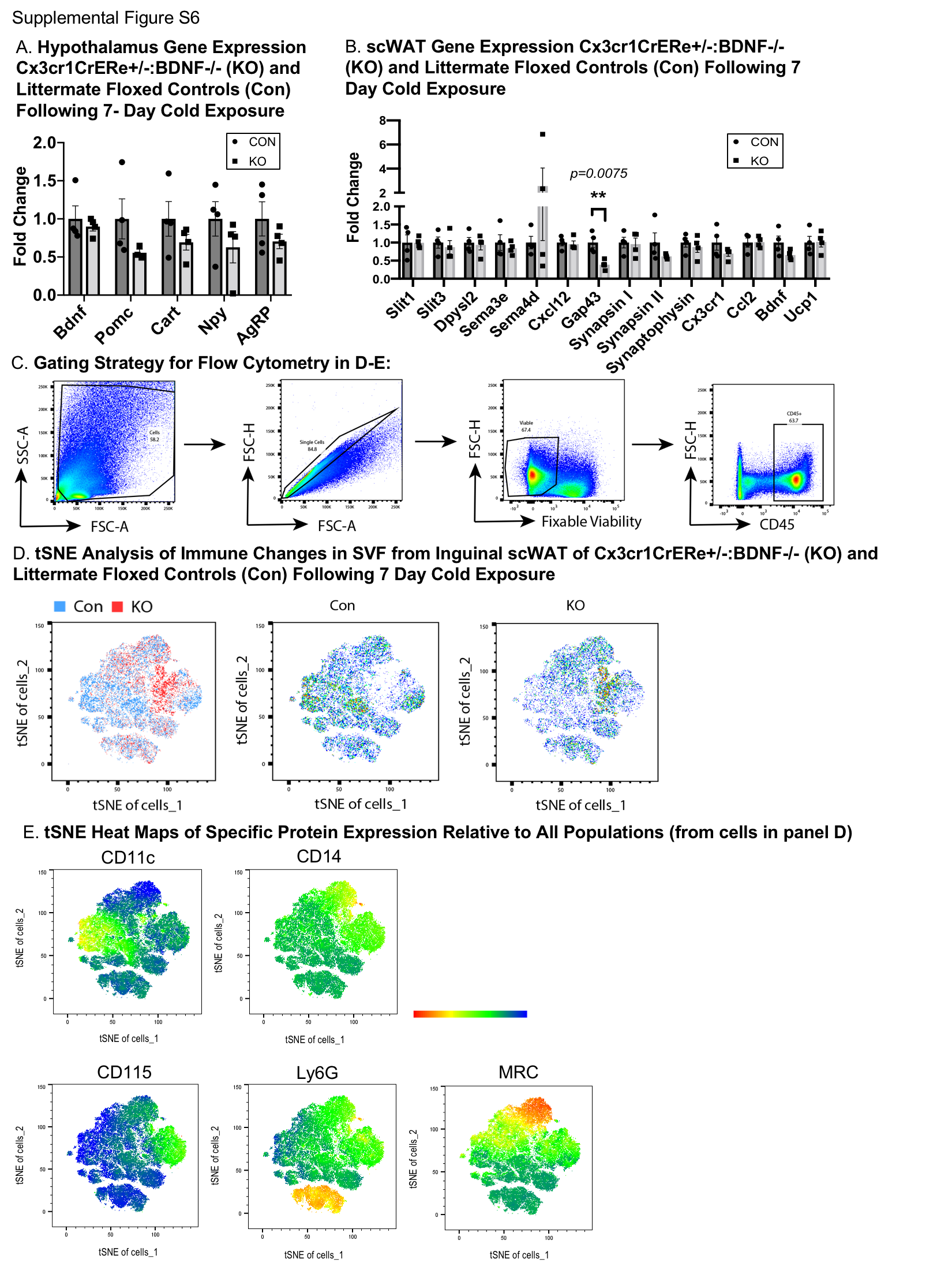

Supplement: Supplementary file 7 [file Image_6.tiff]
