# Supplementary material for: Adipose Tissue Myeloid-Lineage Neuroimmune Cells Express Genes Important for Neural Plasticity and Regulate Adipose Innervation
Source: Front Endocrinol (Lausanne). 2022 Jun 20;13:864925. doi: 10.3389/fendo.2022.864925 (PMC9251313; doi:10.3389/fendo.2022.864925)
Supplement: Supplementary file 8 [file Table_1.docx]

| **Top 10 DE genes by q-value** | | | | | | | |
| --- | --- | --- | --- | --- | --- | --- | --- |
| **Upregulated** | | | | **Downregulated** | | | |
| **Gene symbol** | **q value** | **logFC** | **Entrez Gene Name** | **Gene symbol** | **q value** | **logFC** | **Entrez Gene Name** |
| Add3 | 9.78E-05 | 1.56223768 | Adducin 3 | Usp40 | 0.00016623 | -1.574714 | Uniquitin specific peptidase 40 |
| Dpysl2 | 0.00025508 | 1.1783934 | Dihydropyrimidinase like 2 | Slc7a11 | 0.00016623 | -1.6448062 | Solute carrier family 7 member 11 |
| Cotl1 | 0.00028749 | 1.11612228 | Coactosin like F-actin binding protein 1 | Mroh2a | 0.00021419 | -2.0262314 | Maesto heat like repeat family member 2A |
| Sdc3 | 0.00031431 | 1.08372513 | Syndecan 3 | Kif24 | 0.00025192 | -1.6574341 | Kinesin family member 24 |
| Olfm1 | 0.0003161 | 1.33588693 | Olfactomedin 1 | Tk1 | 0.00026272 | -1.598556 | Thymidine kinase 1 |
| Rac2 | 0.00035784 | 1.14622736 | Rac family small GTPase 2 | Axdnd1 | 0.00035784 | -1.7827986 | Axonemal dynein light chain domain containing 1 |
| Tagln2 | 0.00041957 | 1.28621522 | Transgelin 2 | Proz | 0.00042237 | -1.3400726 | Protein Z, vitamin K dependent plasma glycoprotein |
| Tppp3 | 0.00042237 | 1.75897153 | Tubulin polymerization promoting protein family member 3 | Atp2b2 | 0.0004762 | -2.2401366 | ATPase plasma membrane Ca2+ transporting 2 |
| Fam234a | 0.00049184 | 1.19419639 | Family with sequence similarity 234 member A | Gclc | 0.00056744 | -1.4704659 | Glutamate-cysteine ligase catalytic subunit |
| C5ar1 | 0.00054091 | 1.39666778 | Complement C5a receptor 1 | Rmc1 | 0.00058053 | -1.0762877 | Regulator of MON1-CCZ1 |

Supplemental Table 1

| **Top 10 DE genes by LogFC** | | | | | | | |
| --- | --- | --- | --- | --- | --- | --- | --- |
| **Upregulated** | | | | **Downregulated** | | | |
| **Gene symbol** | **q value** | **logFC** | **Entrez Gene Name** | **Gene symbol** | **q value** | **logFC** | **Entrez Gene Name** |
| Fcna | 0.0689 | 2.572 | ficolin A | Mpig6b | 0.00116 | -3.082 | megakaryocyte and platelet inhibitory receptor G6b |
| MAMDC2 | 0.00471 | 2.518 | MAM domain containing 2 | Ugt1A6 | 0.000678 | -2.96 | UDP glucuronosyltransferase family 1 member A6 |
| Chil3/Chil4 | 0.000589 | 2.194 | chitinase-like 3 | Grm4 | 0.00103 | -2.915 | glutamate metabotropic receptor 4 |
| FAM78B | 0.0115 | 2.177 | family with sequence similarity 78 member B | Ugt1A4 | 0.00162 | -2.904 | UDP glucuronosyltransferase family 1 member A4 |
| CHRM3 | 0.0318 | 2.04 | cholinergic receptor muscarinic 3 | Hipk4 | 0.00189 | -2.849 | homeodomain interacting protein kinase 4 |
| NPL | 0.00968 | 2.039 | N-acetylneuraminate pyruvate lyase | Lman1L | 0.00968 | -2.803 | lectin, mannose binding 1 like |
| CCR4 | 0.0411 | 1.995 | C-C motif chemokine receptor 4 | Lsm7 | 0.000773 | -2.637 | LSM7 homolog, U6 small nuclear RNA and mRNA degradation associated |
| LYVE1 | 0.0765 | 1.98 | lymphatic vessel endothelial hyaluronan receptor 1 | Vwa7 | 0.00258 | -2.621 | von Willebrand factor A domain containing 7 |
| Akr1c12/Akr1c13 | 0.03 | 1.895 | aldo-keto reductase family 1, member C13 | Kcnn | 0.00325 | -2.593 | potassium calcium-activated channel subfamily N member 1 |
| SCN1B | 0.0126 | 1.867 | sodium voltage-gated channel beta subunit 1 | Ermap | 0.00347 | -2.579 | erythroblast membrane associated protein (Scianna blood group) |
