# Supplementary material for: Adipose Tissue Myeloid-Lineage Neuroimmune Cells Express Genes Important for Neural Plasticity and Regulate Adipose Innervation
Source: Front Endocrinol (Lausanne). 2022 Jun 20;13:864925. doi: 10.3389/fendo.2022.864925 (PMC9251313; doi:10.3389/fendo.2022.864925)
Supplement: Supplementary file 9 [file Table_2.docx]

Supplemental Table S2

| **Genes in Top KEGG Pathway Upregulated in Cold Stimulated CINCs: Pathways of Neurodegeneration** | | | | | | | |
| --- | --- | --- | --- | --- | --- | --- | --- |
| **Gene Symbol** | **logFC** | **q value** | **ENTREZID** | **Gene Symbol** | **logFC** | **q value** | **ENTREZID** |
| Mapk12 | -2.2406043 | 0.00212124 | 29857 | ND1 | 0.61628241 | 0.00893772 | 17716 |
| Fzd9 | -2.1838559 | 0.00972702 | 14371 | Csnk2b | 0.62760566 | 0.01159029 | 13001 |
| Dnal1 | -1.679866 | 0.0017871 | 105000 | Ube2l3 | 0.62950345 | 0.02446939 | 22195 |
| Wnt4 | -1.6404108 | 0.00991349 | 22417 | Ndufb10 | 0.64491254 | 0.03952975 | 68342 |
| Atp2a1 | -1.5599709 | 0.02184902 | 11937 | Ndufa6 | 0.64635521 | 0.04262886 | 67130 |
| Ager | -1.5127916 | 0.03879994 | 11596 | Atp5b | 0.65339363 | 0.00967576 | 11947 |
| Grin2d | -1.4830003 | 0.0217579 | 14814 | Map2k7 | 0.65760876 | 0.028879 | 26400 |
| Mapt | -1.436411 | 0.00370822 | 17762 | Psma4 | 0.66977247 | 0.00961991 | 26441 |
| Ryr1 | -1.430139 | 0.00162137 | 20190 | Lrp5 | 0.68488188 | 0.00746254 | 16973 |
| Wnt2b | -1.4234375 | 0.04338876 | 22414 | Atf4 | 0.6857185 | 0.00253349 | 11911 |
| Il1a | -1.4173329 | 0.01761463 | 16175 | Cat | 0.7051247 | 0.00859943 | 12359 |
| Mapk11 | -1.1258085 | 0.02934454 | 19094 | Klc4 | 0.71826365 | 0.04102577 | 74764 |
| Raf1 | -0.5846846 | 0.02498148 | 110157 | Ndufa10 | 0.75559663 | 0.03339538 | 67273 |
| Ern1 | -0.5077301 | 0.01650904 | 78943 | Uqcrfs1 | 0.77366754 | 0.01776035 | 66694 |
| Dvl1 | -0.494722 | 0.0408663 | 13542 | App | 0.78901722 | 0.00304935 | 11820 |
| Dvl2 | -0.4920513 | 0.03707262 | 13543 | Gpx1 | 0.78910697 | 0.00088539 | 14775 |
| Atg2b | -0.4246046 | 0.03460037 | 76559 | Sdhb | 0.80327321 | 0.01595218 | 67680 |
| Ctnnb1 | 0.33634531 | 0.04091494 | 12387 | Park7 | 0.81986223 | 0.01268233 | 57320 |
| Rab1a | 0.35334935 | 0.04418514 | 19324 | Tubb2a | 0.84288856 | 0.02905279 | 22151 |
| Atp2a2 | 0.38541246 | 0.02600427 | 11938 | Ndufs2 | 0.84496086 | 0.00685821 | 226646 |
| Kif5b | 0.39590276 | 0.02502512 | 16573 | Sdha | 0.85117776 | 0.00116936 | 66945 |
| Actr1a | 0.3993062 | 0.04914006 | 54130 | Atp5a1 | 0.85979073 | 0.00113421 | 11946 |
| Slc25a5 | 0.41448126 | 0.04692269 | 11740 | Mapk14 | 0.87086742 | 0.00097217 | 26416 |
| Map3k5 | 0.41721522 | 0.02134141 | 26408 | Cox8a | 0.87658953 | 0.01494481 | 12868 |
| Vcp | 0.41838157 | 0.0276335 | 269523 | Ndufa8 | 0.89274794 | 0.0478755 | 68375 |
| Pik3c3 | 0.45677813 | 0.0464136 | 225326 | Capn1 | 0.90183865 | 0.00459861 | 12333 |
| Atf6 | 0.46391484 | 0.02923327 | 226641 | Ppp3ca | 0.90449951 | 0.0008519 | 19055 |
| Ppp3r1 | 0.47498315 | 0.03053598 | 19058 | Ndufa9 | 0.95822093 | 0.03404529 | 66108 |
| Csnk1e | 0.47723949 | 0.03167359 | 27373 | Uqcrc1 | 0.98399934 | 0.00175484 | 22273 |
| CYTB | 0.49166391 | 0.03603712 | 17711 | Pink1 | 0.99236614 | 0.00354408 | 68943 |
| Prkcb | 0.49468706 | 0.01663034 | 18751 | Nrbf2 | 0.99635419 | 0.0377403 | 641340 |
| Rela | 0.49543942 | 0.01373138 | 19697 | Slc25a4 | 1.00015792 | 0.00316446 | 11739 |
| Ndufa13 | 0.51493602 | 0.04392087 | 67184 | Vdac1 | 1.07937834 | 0.00362502 | 22333 |
| Map2k1 | 0.52963505 | 0.02503847 | 26395 | Cox6a1 | 1.13637471 | 0.00421136 | 12861 |
| Tubb5 | 0.54697644 | 0.01417899 | 22154 | Capn2 | 1.15854596 | 0.00099049 | 12334 |
| Ubc | 0.55757049 | 0.0157311 | 22190 | Sdhc | 1.16461605 | 0.00535217 | 66052 |
| Ube2j1 | 0.57784385 | 0.0168553 | 56228 | Gpx3 | 1.3156862 | 0.01079935 | 14778 |
| Tnfrsf1a | 0.58619195 | 0.01578147 | 21937 | Uqcr11 | 1.32523815 | 0.02636163 | 66594 |
