# Supplementary material for: Adipose Tissue Myeloid-Lineage Neuroimmune Cells Express Genes Important for Neural Plasticity and Regulate Adipose Innervation
Source: Front Endocrinol (Lausanne). 2022 Jun 20;13:864925. doi: 10.3389/fendo.2022.864925 (PMC9251313; doi:10.3389/fendo.2022.864925)
Supplement: Supplementary file 10 [file Table_3.docx]

Supplemental Table S3

| **Network #1 Cellular Assembly and Organization, Cellular Function and Maintenance, Tissue Development** | | | |
| --- | --- | --- | --- |
| **Diseases or Functions Annotation** | **p-value** | **Molecules in Dataset** | **# Molecules** |
| Axonogenesis | 0.0438 | ACTR3,ARHGDIA,Cdc42 | 3 |
| Branching of axons | 0.0303 | ACTR3,Cdc42 | 2 |
| Branching of hippocampal neurons | 0.0253 | Cdc42 | 1 |
| Branching of neurites | 0.000871 | ACTR3,Cdc42,CFL1,DBN1,PFN1,TMOD1 | 6 |
| Dendritic growth/branching | 0.0123 | CFL1,DBN1,PFN1,TMOD1 | 4 |
| Density of dendritic spines | 0.0419 | PFN1,TMOD1 | 2 |
| Density of mushroom spine | 0.0189 | TMOD1 | 1 |
| Density of neurons | 0.0136 | DBN1,PFN1,TMOD1 | 3 |
| Extension of axons | 0.00334 | ACTR3,DBN1,TWF2 | 3 |
| Formation of fiber tract | 0.00277 | Cdc42 | 1 |
| Recruitment of microglia | 0.0358 | Cdc42 | 1 |
