# Supplementary material for: Adipose Tissue Myeloid-Lineage Neuroimmune Cells Express Genes Important for Neural Plasticity and Regulate Adipose Innervation
Source: Front Endocrinol (Lausanne). 2022 Jun 20;13:864925. doi: 10.3389/fendo.2022.864925 (PMC9251313; doi:10.3389/fendo.2022.864925)
Supplement: Supplementary file 11 [file Table_4.docx]

Supplemental Table S4

| **qPCR Primer List** |  |  |
| --- | --- | --- |
| **Gene** | **Forward Sequence 5' -> 3'** | **Reverse Sequence 5' -> 3'** |
| Agrp | ATG CTG ACT GCA ATG TTG CTG | TGA GGC CAT TCA GAC TTA GAC |
| Bdnf | CAG GTG AGA AGA GTG ATG ACC | ATT CAC GCT CTC CAG AGT CCC |
| Cart | ATC TAC TCT GCC GTG GAT | TCC CTC ACT GCG CAC TGC TGT |
| Ccl2 | CCC AAT GAG TAG GCT GGA GA | CCT TAG GGC AGA TGG TGT TC |
| Cxcl12 | CAG TGA CGG TAA ACC AGT CAG C | TGG CGA TGT GGC TCT CG |
| Cx3cr1 | GTT ATT TGG GCC ACA TTG TGG C | CAG ACC GAA CGT GAA GAC GAG |
| Dpysl2 | CTG ACC AGG GAA TGA CAT CC | GAG GCT GGA GTG ACT GTC TT |
| Gap43 | ATA ACT CCC CGT CCT CCA AGG | GTT TGG CTT CGT CTA CAG CGT |
| Npy | AAG CCG GAC AAT CCG GGC CGA | GCT TTC CTC ATT AAG AGG TCT |
| Ppia | GGC AAA TGC TGG ACC AAA C | CAT TCC TGG ACC CAA AAC G |
| Pomc | AGA CCT CCA TAG ATG TGT GGA | AGC GGA AGT GAC CCA TGA CGT |
| Sema3b | GAG TGA GAA CCC TGA TGA CG | CAT TTG TTG ACC AAG CTC CG |
| Sema3e | GGG GCA GAT GTC CTT TTG A | AGT CCA GCA AAC AGC TCA TTC |
| Sema4d | CCT GGT GGT AGT GTT GAG AAC | GCA AGG CCG AGT AGT TAA AGA T |
| Slit1 | CTG CTC CCC GGA TAT GAA CC | TAG CAT GCA CTC ACA CCT GG |
| Slit3 | AGT TGT CTG CCT TCC GAC AG | TTT CCA TGG AGG GTC AGC AC |
| Synapsin I | CAT GGC ACG TAA TGG AGA CTA CCG CA | CCG CCA GCA TGC CTT C |
| Synapsin II | GCC ACC AGG TTA AGC TCT GA | TTC CAG GAA GGC CAA GGT |
| Ucp1 | AGG CTT CCA GTA CCA TTA GGT | CTG AGT GAG GCA AAG CTG ATT T |
| Synaptophysin | TGA CTT CAG GAC TCA ACA CCT C | CAG GAG CTG GTT GCT TTT CT |
| Vasp | ACC CCA CTG CTA ACT CCT TC | GCA AAC TGT ATG GCG TCC TC |
