# Supplementary material for: Adipose Tissue Myeloid-Lineage Neuroimmune Cells Express Genes Important for Neural Plasticity and Regulate Adipose Innervation
Source: Front Endocrinol (Lausanne). 2022 Jun 20;13:864925. doi: 10.3389/fendo.2022.864925 (PMC9251313; doi:10.3389/fendo.2022.864925)
Supplement: Supplementary file 12 [file Table_5.docx]

**Supplemental Figure Legends**

**Supplemental Figure. S1: Hierarchal clustering, Gene Ontogeny(GO) and selected differentially expressed genes from RNAseq of room temperature (RT) versus cold stimulated CINCs. (A)** Unbiased hierarchical clustering of samples used in differential expression analysis. **(B)** Gene Ontogeny (GO) results for the top 10 biological processes (BP) terms associated with cold stimulated (bars to the right) and room temperature (bars to the left) CINCs. **(C)** GO results for the top 10 cellular components (CC) terms associated with cold stimulated (bars to the right) and room temperature (bars to the left) CINCs. **(D)** GO results for the top 10 molecular functions (MF) terms associated with cold stimulated (bars to the right) and room temperature (bars to the left) CINCs. **(E)** Normalized RNA counts of selected differentially expressed (DE) genes related to axonal guidance signaling. **(F)** Normalized RNA counts of selected differentially expressed (DE) genes related to neurotrophin signaling.

**Supplemental Figure. S2: IPA Pathways and Functions predicted in cold stimulated CINCs. (A)** Selected nerve-related pathways activated or inhibited in cold stimulated CICNs compared to room temperature CINCs. Positive z-score predicts activated pathway, negative z-score predicts inhibited pathway (5% FDR threshold was applied to DE genes and a Z score (-2.0 ≤ Z ≥ 2.0) was considered significant). **(B)** Selected nerve related biological functions shown as a network predicted from all differentially expressed genes in the dataset. Center node represents biological function, neuronal cell death (left) and neuritogenesis (right). Decreased neuronal cell death is a predicted function from the differential expression of 414 molecules within the dataset (z-score = -2.131, *p=1.00^-21^*); Neuritogenesis was a predicted function of CINCs (z-score 0.440, p=1.61^-21^) based on 379 molecules within the dataset. Legends show activation/inhibition relationships, types of molecule within the network and level of DE.

**Supplemental Figure S3: IPA Network Analysis.** All DE genes in the dataset were used to generate molecular networks using IPA. Networks are in order of score, from most to least, significant, corresponding to the p value calculated for the network determined via right-tailed Fischer’s exact test, p values for each network are listed in Supplemental Table S3. Top Biological Function and Disease for each network are as follows **(A)** Network #2: Cellular Morphology, Cellular Assembly and Organization, Cellular Function and Maintenance **(B)** Network #3: Developmental Disorder, Hereditary Disorder, Metabolic Disease **(C)** Network #4: Embryonic Development, Gene Expression, Protein Synthesis **(D)** Network #5: Carbohydrate Metabolism, Cell-To-Cell Signaling and Interaction, RNA Post-Transcriptional Modification **(E)** Network #6: Cell Cycle, Infectious Disease, RNA Post-Transcriptional Modification **(F)** Network #7: Cancer, Gastrointestinal Disease, Gene Expression **(G)** Network #8: Nervous System Development and Function, Neurological Disease, Organismal Injury and Abnormalities **(H)** Network #9: Cancer, Cell Cycle, RNA Post-Transcriptional Modification **(I)** Network #10**:** Molecular Transport, RNA Post-Transcriptional Modification. CP=Canonical pathway, Fx=Function, Orange=Leads to activation, Blue=Leads to inhibition, Yellow=Findings inconsistent with state of downstream molecule, Grey=Effect not predicted

**Supplementary Figure S4.** **Whole mount imaging of TrkB expression in T12 DRGs of room temperature and cold exposed mice.** **(A)** Single micrographs and digitally zoomed insets corresponding to tiled whole mount imaging in Fig 3A. **(B)** Single micrographs corresponding to tiled whole mount imaging in Fig 3C. **(C)** Male TrkB^GFP^ reporter mice were housed at room temperature (RT) or cold exposed (at 5°C) for 10 days prior to tissue collection (RT N=3, Cold N=3). Dorsal root ganglia (DRG) at thoracic vertebra 12 (T12) were excised and immunolabeled with anti-GFP and DAPI. Representative images of RT and Cold exposed DRGs. Images were captured by confocal microscopy using a white light laser (WLL) at 10X objective magnification and are displayed as z-maximum intensity projections.

**Supplemental Figure S5. BDNF expression in scWAT. (A)** Adult (19-20 week old) male LysMCre^+/-^:BDNF^-/-^ (KO, N=4)) and littermate LysMCre^+/-^:BDNF^fl/fl^ control (Con, N=4) mice were cold exposed (at 5°C). BDNF expression in the whole scWAT depot was measure by ELISA.

**Supplemental Figure S6. Gene expression in hypothalamus and scWAT; and scWAT immune profile of *Cx3cr1CrERe^+/-^:BDNF^-/-^* (KO) and littermate floxed controls (Con). (A)** Relative gene expression of *Bdnf* and factors related to hypothalamic control of appetite: Proopiomelanocortin (*Pomc*), Cocaine and amphetamine regulated transcript (*Cart*), Neuropeptide Y (*Npy*), and Agouti-related peptide (*Agrp*) in hypothalamus of Con (N=4) and KO mice (N=4). Note: Gene expression is shown as fold change in ΔΔCt values, normalized to Con control mice, for panels A-B. **(B)** Relative gene expression of select axonal guidance and synaptogenesis markers in scWAT of Con (N=4) and KO mice (N=4) following 7-day cold (5°C) exposure. For A and B, qPCR data were analyzed by two-tailed Student’s T-Test. All error bars are SEMs. *p < 0.05, **p < 0.01, ***p < 0.001, ****p < 0.0001. **(C-E)** Adult (15-17 week old) male *Cx3cr1CrERe^+/-^:BDNF^-/-^* (KO) and their littermate control *Cx3cr1CrERe^-/-^::BDNF^fl/fl^* (Con) mice were cold exposed (5°C) for 7 days and SVF from bilateral inguinal scWAT depots was isolated for flow cytometry, N=3 per group. **(C)** Gating strategy for flow cytometric analyses in D-E. **(D)** Representative t-Distributed Stochastic Neighbor Embedding (tSNE) Analysis of immune cells in SVF from inguinal scWAT of Cx3cr1CrERe+/-:BDNF-/- (KO) and littermate floxed controls (Con), showing overlay of Con and KO mice together, and individual tSNE of only Con or KO mice. **(E)** Top panel: tSNE heat map overlays of CD11c (moDCs) and CD14 (activated macrophages) expression relative to all viable CD45+ cells. Bottom panel: tSNE heat map overlays of CD115 (monocyte/macrophages), Ly6G (neutrophils), and MRC (alternatively activated macrophages) relative to all viable CD45+ cells.
